# Beyond the EDGE with EDAM: Prioritising British Plant Species According to Evolutionary Distinctiveness, and Accuracy and Magnitude of Decline

**DOI:** 10.1371/journal.pone.0126524

**Published:** 2015-05-27

**Authors:** William D. Pearse, Mark W. Chase, Michael J. Crawley, Konrad Dolphin, Michael F. Fay, Jeffrey A. Joseph, Gary Powney, Chris D. Preston, Giovanni Rapacciuolo, David B. Roy, Andy Purvis

**Affiliations:** 1 Department of Life Sciences, Imperial College London, Ascot, Berkshire, United Kingdom; 2 Centre for Ecology and Hydrology, Wallingford, Oxfordshire, United Kingdom; 3 University of Minnesota, Department of Ecology, Evolution, and Behavior, 100 Ecology Building, 1987 Upper Buford Circle, Saint Paul, Minnesota 55108 USA; 4 Jodrell Laboratory, Royal Botanic Gardens Kew, Richmond, Surrey TW7 3DS, UK; 5 Department of Life Sciences, Natural History Museum, Cromwell Road, London SW7 5BD, UK; University of Colorado, UNITED STATES

## Abstract

Conservation biologists have only finite resources, and so must prioritise some species over others. The *EDGE*-listing approach ranks species according to their combined evolutionary distinctiveness and degree of threat, but ignores the uncertainty surrounding both threat and evolutionary distinctiveness. We develop a new family of measures for species, which we name *EDAM*, that incorporates evolutionary distinctiveness, the magnitude of decline, and the accuracy with which decline can be predicted. Further, we show how the method can be extended to explore phyogenetic uncertainty. Using the vascular plants of Britain as a case study, we find that the various *EDAM* measures emphasise different species and parts of Britain, and that phylogenetic uncertainty can strongly affect the prioritisation scores of some species.

## Introduction

Global biodiversity is declining [1, 2], forcing conservation biologists to prioritise their finite conservation budgets in order to save as many species as possible (the ‘Noah’s Ark problem’;[3]). The *EDGE* approach [4] prioritises species according to a combination of how evolutionary distinctive (‘*ED*’) and how globally endangered (‘*GE*’) they are. *EDGE* listing is a popular approach (cited over 100 times on *Web of Knowledge*), and has been applied to mammals [4] and amphibians [5], and related methods have been used for birds [6, 7]. Evolutionary distinctiveness is an appealing metric to many biologists [8]; beauty and utility are difficult to quantify, but the millions of years of evolutionary history a species or clade uniquely represents are not.

*EDGE* listing has proven a useful tool, but its components are not optimised for local-scale prioritisation. Red Listing status (the *GE* component) is a global ranking, as its maintainer (IUCN) acknowledges by funding the ‘National Red List’ project (http://www.nationalredlist.org/). Similarly, calculating evolutionary distinctiveness with a global phylogeny will underestimate the national distinctiveness of species with close relatives living only in other countries [9]. Such clades might be distinctive and important local components of many ecosystems, but could paradoxically be prioritised in none of them.

More fundamentally, *EDGE* does not attempt to distinguish between the magnitude of a threat and the extent to which we understand that threat. A species undergoing a large decline that is understood to be a transient part of a natural cycle may be of lesser concern than a species undergoing a smaller decline that we do not understand or know how to reverse. Distinguishing among the finer points of species’ threats is impractical when dealing with the thousands of species in the global Red Lists, but individual countries have fewer species and often have more detailed, comparable within-country data.

We propose a family of prioritisation strategies (‘*EDAM*’) that extends the *EDGE* system, incorporating species’ evolutionary distinctiveness, magnitude of decline, and the accuracy with which we can predict declines in the absence of successful conservation intervention. Each of these components is transformed to be on a common numerical scale, making their implicit trade-off in *EDGE* explicit in *EDAM*. Using a novel genus-level phylogeny for the majority of vascular plant species in Britain, we report the species and parts of Britain that *EDAM* and *EDGE* prioritise.

## *EDAM* framework

*EDGE* consists of two components: evolutionary distinctiveness (‘*ED*’; the phylogenetic diversity of a clade split equally among its members [4], which is related to how much branch length is unique to each species), and how globally endangered (‘*GE*’) a species is according to the IUCN Red List. We propose a general family of prioritisation indices (‘*EDAM*’), which incorporate *ED*, the accuracy with which decline (or threat) can be predicted (‘*A*’), and the magnitude of that decline (or threat) (‘*M*’). There are many ways of assessing all of these components; subscripts can be used to distinguish among them where there is ambiguity, but where there are no subscripts we use precision to measure accuracy, and range change to measure magnitude. We describe the specific evolutionary, accuracy, and magnitude components that we use in this study in the next section, and Table 1 summarises each of them as well. *EDAM* indices, like *EDGE*, are the sum of their components, but under *EDAM* each of these components is scaled to have a mean of zero and standard deviation of one. Thus, each component contributes equally to the measure, and judgements about which components are more important can readily be made explicit by multiplying components by a weighting factor.

**Table 1:** Summary of measures. Note that all measures prioritise evolutionary distinctiveness (‘ED’ above), and that all components are scaled such that their means are zero and standard deviations one unless stated. As discussed in the text, in the *EDAM* framework *EDGE* values could be called *EDM*_*RL*_, although *EDGE* components are not scaled.

Measure	Formula	Prioritises
*EDGE* (*EDM*_*RL*_)	unscaled ln(ED) + Red List	Global threat
*EDM*	ln(ED) + range change	British threat
*EDAM*	ln(ED) + range change + *precision* of decrease	Range change and low *precision* of decrease
*EDAM*′	ln(ED) + reversed range change + *precision* of increase	Range change and low *precision* of increase
*ED*(*AM*)_*max*_	ln(ED) + max(range change, *precision*)	Greatest value of threat and accuracy of prediction

An *EDAM* measure need not contain all three terms. For instance, *EDM*_*RL*_ incorporates only evolutionary distinctiveness and Red Listing status; and differs from *EDGE* only in that its components are scaled. However, $EDM_{RL}^{\prime }$ incorporates evolutionary distinctiveness and the *negation* of Red Listing status (indicated by the prime)—this would prioritise species that are evolutionarily distinct and not threatened. Such species are not of conservation concern, but evolutionarily distinct species with increasing ranges could be potentially damaging invasive species (as discussed with *EDAM*′ in the case study below). If components are to be multiplied by scaling factors, we suggest they are represented in the superscripts of the measures; for example, *EDM* ^2^ would weight the magnitude of decline as twice as important as evolutionary distinctiveness, and vice-versa for *ED* ^2^ *M*. This labelling scheme has been chosen such that superscript signifies multipliers of the quantitative effect of a term, while subscript denotes modifiers that indicate a different kind of measure. There are also *precautionary* measures such as *ED*(*AM*)_*max*_, for which evolutionary distinctiveness (ED) is added to the greatest of the accuracy (A) and magnitude (M) measures, emphasising species that are either declining rapidly, or with range dynamics we understand poorly. Such a measure would not be possible if both accuracy and magnitude had not been transformed to be on a common scale.

Weighting conservation actions according to the confidence we place in the composition of clades in a phylogeny (*e.g.*, bootstrap values) would be unhelpful, but it is useful to know the effect that phylogenetic uncertainty has on prioritisation. Most methods of phylogeny construction produce a set of credible, but not optimal, trees, and repeating analyses across this subset accommodates phylogenetic uncertainty. Estimates could be weighted by the likelihood of the tree across which each measure was calculated, estimating the impact of phylogenetic uncertainty.

## Case study: British vascular plants

*EDAM* measures have three components: evolutionary distinctiveness, accuracy of decline or threat, and magnitude of decline or threat. Below we describe how each of these components was generated, and then how each *EDAM* measure was calculated. For comparison, *EDGE* scores were also calculated. Note that the *EDAM* results are based on decline, whereas the Red Listing data upon which the *EDGE* scores are based somewhat blur the distinction between decline and threat depending upon the criterion under which a species is listed [10].

### Phylogeny building (evolutionary distinctiveness)

Samples from 548 species were collected from natural British populations, each representing a different plant genus. All samples were collected as part of the UK Flora DNA Bank Project, under licence from Natural England. The *rbcL* locus was amplified either as a single fragment, or two overlapping, PCR product(s) to obtain the whole region. Ten primers were utilised in a number of different combinations to obtain *rbcL* sequences from diverse groups of plant taxa. Eight of these primers were from previous publications: 1F, 1460R (5′ TCC TTT TAG TAA AAG ATT GGG CCG AG 3′), 724R, 636F [11]; 724Rm [12]; 1360R [13]; 32F, 1367R [14]. The remaining primers were developed at RBG Kew (Genetics Section): 1FA (5′ ATG TCA CCA CAA ACA GAG AC 3′) and 627F (5′ CAT TTA TGC GCT GGA GAG ACC G 3′).

Double stranded PCR amplification of *rbcL* was performed in an ABI thermal cycler, using pre-made 2.5mM MgCl_2_ PCR Mastermix (ABgene), 14*μ*M of forward and reverse primer, 1.0*μ*l BSA (0.4% w/v), and between 50–100ng of total DNA, in 50*μ*l reaction volumes. Thermal cycler conditions were (1) 96°C, 1min; (2) 96°C, 1min, (3) 48°C, 30sec, (4) 72°C, 1min; cycle (2)-(4) was repeated for 28 cycles, (5) 72°C, 7min; (6) 4°C. Products were cleaned using QIAquick PCR Purification Kit (Qiagen) according to the manufacturer’s protocol. These DNA fragments were sequenced using 2.0*μ*l BigDye^™^ ABI PRISM^™^ (Perkin-Elmer), 10–20ng of PCR DNA, and either 5′ forward or 3′ reverse primer (1.4*μ*M) in 5.25*μ*l reaction volumes. ABI thermal cycler conditions for dye-terminating reactions were 96°c, 1min; 66°c, 5sec; 72°c, 45sec; for 26 cycles. The sequencing products were analysed on an ABI 3100 automated DNA Sequencer (Perkin-Elmer). Both 5′ (forward) and 3′ (reverse) DNA sequences were obtained from each PCR product, and assembled and edited using Autoassembler (Version 1.4.0).

All samples sequenced as above have been released onto *GenBank* [15] (see S1 File for accession numbers), but only 364 of them were used for this project. An additional 312 different *rbcL* sequences were downloaded from *GenBank* using *phyloGenerator* [16], meaning the alignment altogether represents 91% of native genera in Britain (according to *PLANTATT*; [17]). We constructed a family-level constraint tree based on the APG III classification [18] included with *Phylomatic* [19]. After these analyses were performed, fourteen of the sequences were found to have sequencing errors that caused frame-shifts at the extreme 3′ end of the sequences. The corrected sequences were uploaded to GenBank, but the uncorrected sequences, along with a demonstration that these sequence-changes do not alter phylogenies built with them, are presented in S1 File.

We aligned the sequences using *MAFFT* [20, 21], and chose the phylogeny with the greatest likelihood (under a *GTR*-*γ* DNA substitution model) from two separate *RAxML* [22] runs. Each run partitioned the alignment into three codon positions, used the ‘*GTR-PSR*’ (previously called ‘*GTR-CAT*’) DNA substitution model, and was constrained using the constraint tree described above. The first run used 500 random starting trees (the log likelihood of the best tree was −93656.22), and the second was an integrated rapid bootstrap search with 2000 random bootstrap searches and 400 subsequent thorough maximum likelihood searches (the log likelihood of the best tree was −93669.50). Since the best-scoring tree was found in the first search, we annotated that tree with the 2000 rapid bootstrap trees from the second search, and rate smoothed it using *PATHd8* [23], setting the root age to 1.

Genera in the phylogeny containing more than one species were replaced with a polytomy containing all the species listed in *PLANTATT* in that genus, with the polytomy placed either half-way along the branch that led to the representative of that genus in the phylogeny, or at the 80^*th*^ quantile of genus age in the phylogeny, whichever was smaller. This reduces bias introduced by particularly isolated sister species; many of the gymnosperm genera are distantly related to one another, and excessively long branches within genera could have biased the results. While more complex algorithms are available to assign ages to polytomies (*e.g.*, [24]) the quantile at which the cut is made does not affect these results (see S1 Fig), and it is practical to apply this method to large numbers of phylogenies (see below).

Evolutionary distinctiveness was calculated across the phylogeny using the *ed.calc* function in *caper* [25] with the *Isaac* correction for polytomies. The natural logarithm of these *ED* values was used to calculate *EDGE*, and the same (log-transformed) data were scaled to have a mean of 0 and standard deviation of 1 for use in the *EDAM* measures. In the original *EDGE* list 1 was added to the *ED* values before taking their logarithm to normalise the result [4]; doing so was not necessary in this case since the data were normally distributed, and in fact would have made the data extremely non-normally distributed. The entire procedure of rate smoothing, species addition, and *ED* calculation was additionally performed using each of the 2000 phylogenies produced during the rapid bootstrap search.

### Magnitude of decline or threat

We used the Telfer *et al.* [26] relative change index (RCI) to measure species decline, using the same methods as in [27]. Briefly, RCI was defined for each species as the residuals from a regression of the logit-transformed proportion of occupied 10km × 10km cells in 1987–1999 (taken from [28]) against the logit-transformed proportion of occupied cells in 1930–1969 (taken from an update to [29]). RCI is not an absolute measure of decline; the observed decline is relative to the other species in the dataset, but for the purpose of prioritisation this distinction is unimportant. Range change values were reversed so that greater numbers indicate a greater decline, and scaled to have a mean of 0 and standard deviation of 1. When calculating *EDGE*, Red List category (taken from the 2011 update of [30]) was treated as a continuous variable on a coarse scale (Least Concern = 0, Near Threatened and Conservation Dependent = 1, Vulnerable = 2, Endangered = 3, Critically Endangered = 4; as in [4]).

### Accuracy of decline

Correlative species distribution models (SDMs) predict species’ potential range expansions and contractions based on the redistribution of the species’ realised environment over time and/or space [31]; the predictive accuracy of SDMs in new environmental domains is therefore a good measure of how well species’ declines may be predicted based on given environmental variables. We modelled distribution data at the 10km square resolution for each plant species in the period 1930–1969 as a function of climate and land use using generalised boosted models (GBMs; [32]); we then quantified the predictive accuracy of models by comparing model forecasts to independent observations for the period 1987–1999. We used the same underlying data and methods as [33], but in addition accounted for historic change in land use using data from [34] for the 1930–1969 period and [35] for 1987–1999 period.

The predictive accuracy of SDMs over time is generally calculated by comparing model predictions with independent observations using widespread discrimination measures such as the AUC (*e.g.*, [36, 37]). Here, however, we use one of three new measures of temporal validation for SDMs—*Acc*_*TV*_ [38]—which has two main advantages over most commonly-used alternatives: it makes use of modelled probabilities of presence over time directly, without requiring the choice of arbitrary probability thresholds, and it focuses on accuracy over portions of species’ ranges that have been either observed or predicted to change, thus quantifying a model’s ability to predict decline. Briefly, *Acc*_*TV*_ can be derived from temporal validation (TV) plots, which are extensions of presence-absence calibration plots [39] for use with data from two time periods. TV plots model observed gains and losses as a function of changes in modelled probability of presence between time periods using natural splines (see Rapacciuolo *et al.* [38] for detailed methods). Ideally, summing interpolated gains and losses across values of change in modelled probability of presence should result in an ideal TV curve: a line with a slope of 1, passing through the origin (0, 0). *Acc*_*TV*_ is the weighted average distance between the model’s and the ideal TV curve, subtracted from 1. It can be calculated using the following formula:

$$\begin{array}{c} Acc_{TV}=1-\frac{\sum_{q=1}^{n}\Delta m_{weighted,q}\left|y_{model,q}\right|}{\sum_{q=1}^{n}\Delta m_{weighted,q}} \end{array}$$ (1)

where *y*_*model*_ and *y*_*ideal*_ are the *y* values of the model curve and ideal curve, respectively, at each observed site *q*, and *x*_*q*_ are the proportional changes in modelled probability of presence at each site *q*. Species’ *Acc*_*TV*_ estimates were scaled to have a mean of zero and a standard deviation of one.

### Calculating the measures

We calculated the measures listed in Table 1 using the range change and predictive accuracy data described above, and evolutionary distinctiveness values from the best-scoring phylogenetic tree. We compared *EDGE* and *EDM* by regressing them against each other and their components; all components of *EDAM* were uncorrelated with each other (all ∣*r*∣ < 0.11). Additionally, we calculated *EDM* across all 2000 rapid bootstrap trees, to assess the impact of phylogenetic uncertainty on its values.

Although the various measures may rank the importance of species differently, many conservation efforts are targeted at the habitat-level and so this variation might be of little importance if prioritised species frequently co-occur. To see whether the indices prioritised different parts of Britain, we map the top-fifty ranked species according to each index of the species within each grid cell of the Preston *et al.* distribution data for Britain [28]. Means of prioritisation scores are difficult to interpret due to shifts in species richness across Britain; however, maps of this mean richness are available in S2 Fig.

## Results

### Phylogeny

All novel *rbcL* sequences have been uploaded to *GenBank* [15], and the accession numbers of all sequences used are given in S1 File. The optimal phylogeny and the bootstrap phylogenies are available in S1 File (all rate-smoothed and in Newick format), as is a *OneZoom* [40] file that allows the tree to be interactively explored.

### Comparing *EDGE* and *EDM*_*RC*_

**Fig 1:**
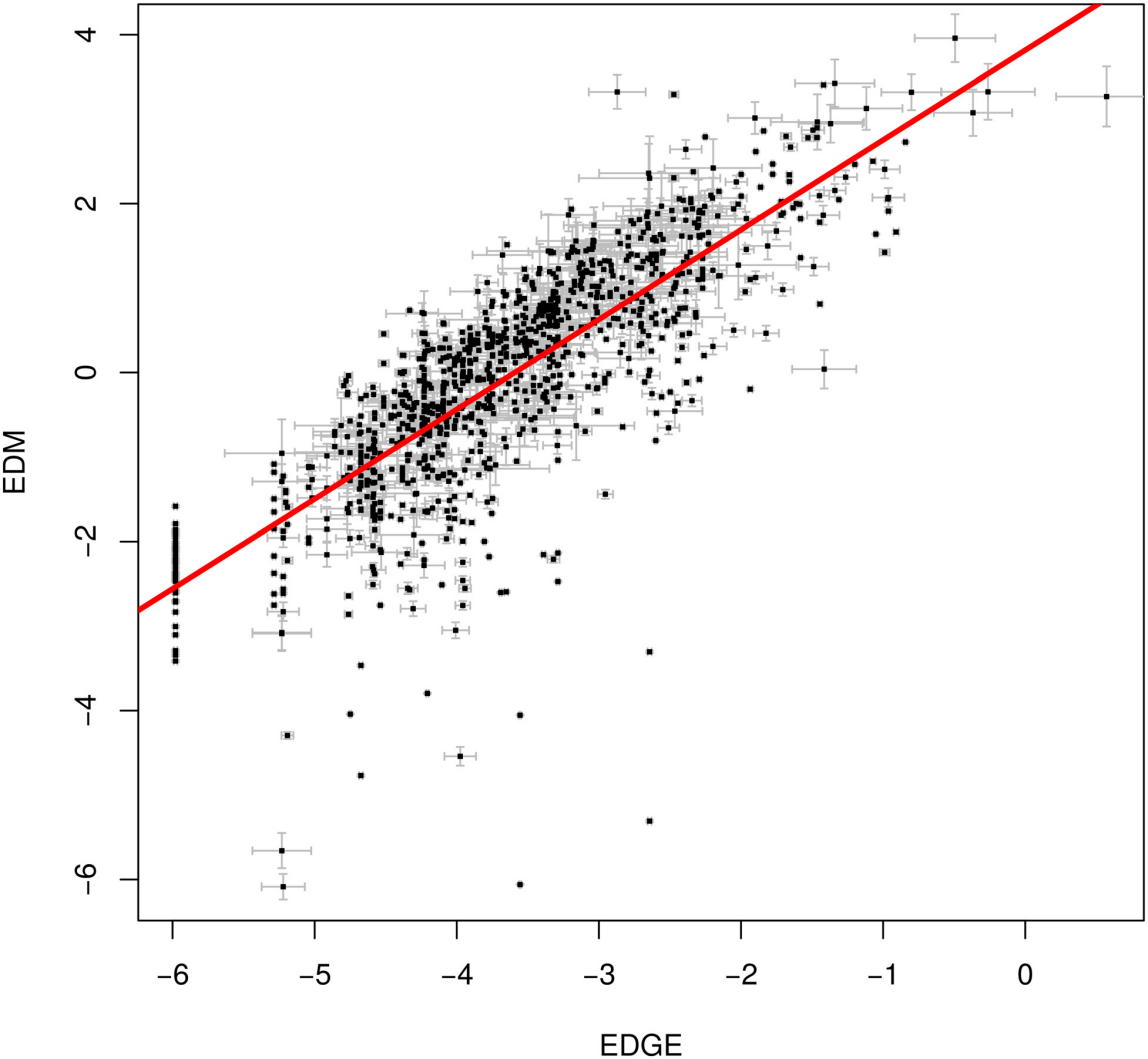
Plot of *EDGE* scores against *EDM*, showing they are strongly correlated (red regression line; *F*_1,1051_ = 1846, *r* ^2^ = 0.64, *p* < 0.0001). The standard deviations of the *EDM* and *EDGE* values’ bootstrapped estimates are shown as grey whiskers around each point; these were not incorporated in the regression quoted above. There are 553 tied *EDGE* scores, and no tied *EDM* scores; a line of tied *EDGE* species can be seen at the left of the plot. The greater number of tied *EDGE* species suggest *EDGE* is worse at discriminating among species.

**Fig 2:**
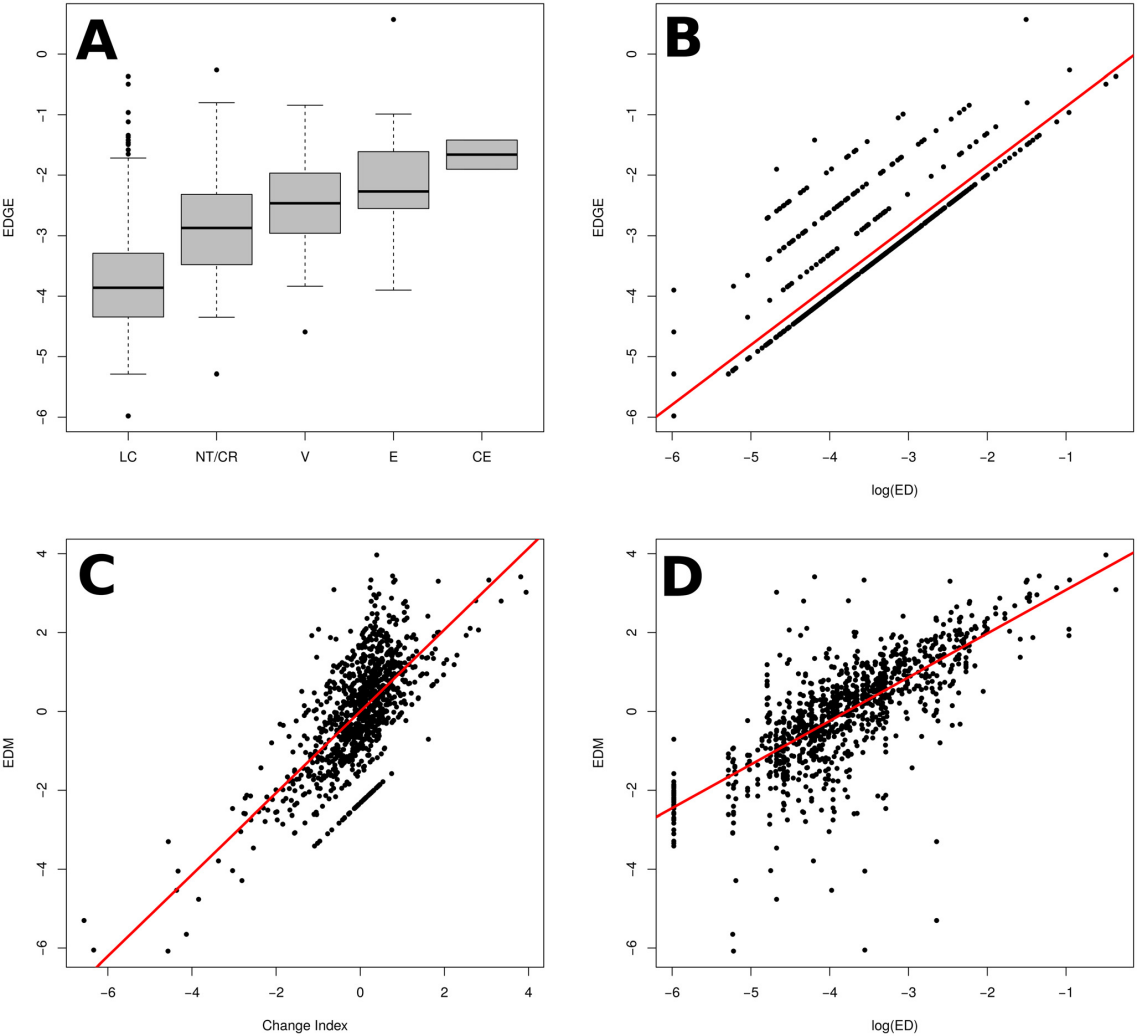
*EDGE* and EDM plotted against their components. In (A) and (B), *EDGE* is plotted against Red List category (increasing in threat level from left-to-right; *r* ^2^ = 0.20) and the logarithm of evolutionary distinctiveness (*r* ^2^ = 0.78) respectively. The larger *r* ^2^ when regressed against log(ED) suggests *EDGE* is more strongly related to *ED* than *GE*. (C) and (D) show *EDM* against change index (*r* ^2^ = 0.47) and the logarithm of evolutionary distinctiveness (*r* ^2^ = 0.56) respectively. *EDM* is related almost equally strongly to its components, and so is less biased than EDGE. All models above were statistically significant (*p* < 0.0001) and were linear regressions, with the exception of comparison of *EDGE* and Red Lists status, which was an ANOVA. Note that *EDM* is calculated with scaled values, but is not regressed against them above.

*EDGE* and *EDM* were strongly correlated, but many more species were tied for *EDGE* values (Fig 1), suggesting that *EDGE* was less discriminating (reflecting the coarser nature of the Red List data). Both measures were significantly correlated with their components, but *EDGE* was more correlated with *ED* than Red Listing status (Fig 2a and 2b), suggesting *EDGE* is driven by *ED* in this dataset. *EDM* was correlated almost equally with *ED* and range change (Fig 2c and 2d), confirming that it reflects its two components equally.

**Fig 3:**
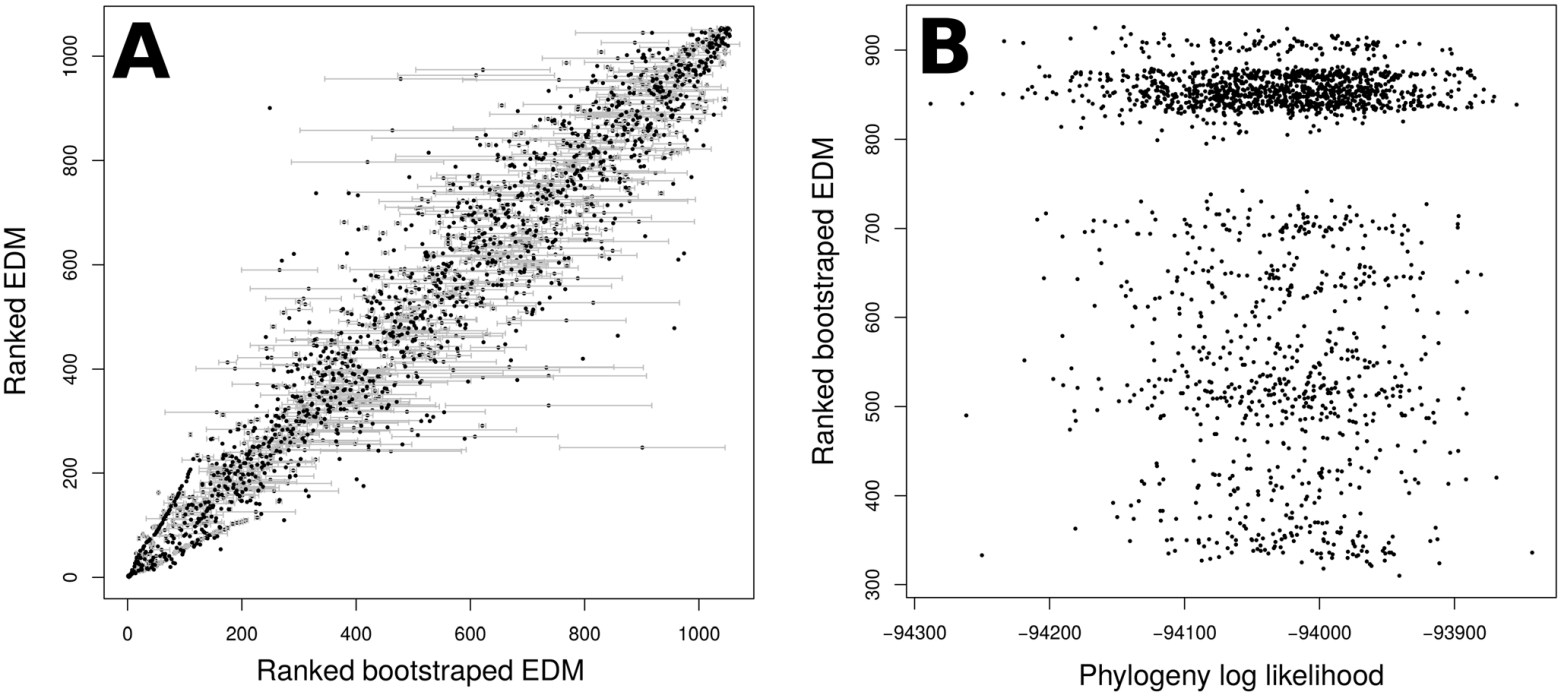
Variability of *EDM* values across bootstrap phylogenies. In (A), the ranks of the best phylogeny’s *EDM* values are plotted against the median ranks across all the bootstrap phylogenies, with grey whiskers showing the standard deviations of those ranks. (B) plots the rank of *EDM* for *Wahlenbergia hederacea* in each bootstrap phylogeny against the log. likelihood of that phylogeny. *Wahlenbergia hederacea* is the species with the largest standard deviation in (A). Three distinct groupings of *EDM* values can be seen in (B), probably reflecting equally likely islands in treespace (see discussion).

The *EDAM* values were stable across the bootstrap phylogenies. All sets of *EDAM* values calculated using the bootstrapped phylogenies, when compared with the *EDAM* values from the optimal phylogeny, had correlation coefficients greater than 0.92. However, Fig 3a reveals *EDM* estimates for some species vary considerably across the bootstraps. The species with the greatest standard deviation of bootstrapped *EDM* is shown in Fig 3b, where three distinct groupings of *EDM* values can be seen.

### Conservation priorities

**Fig 4:**
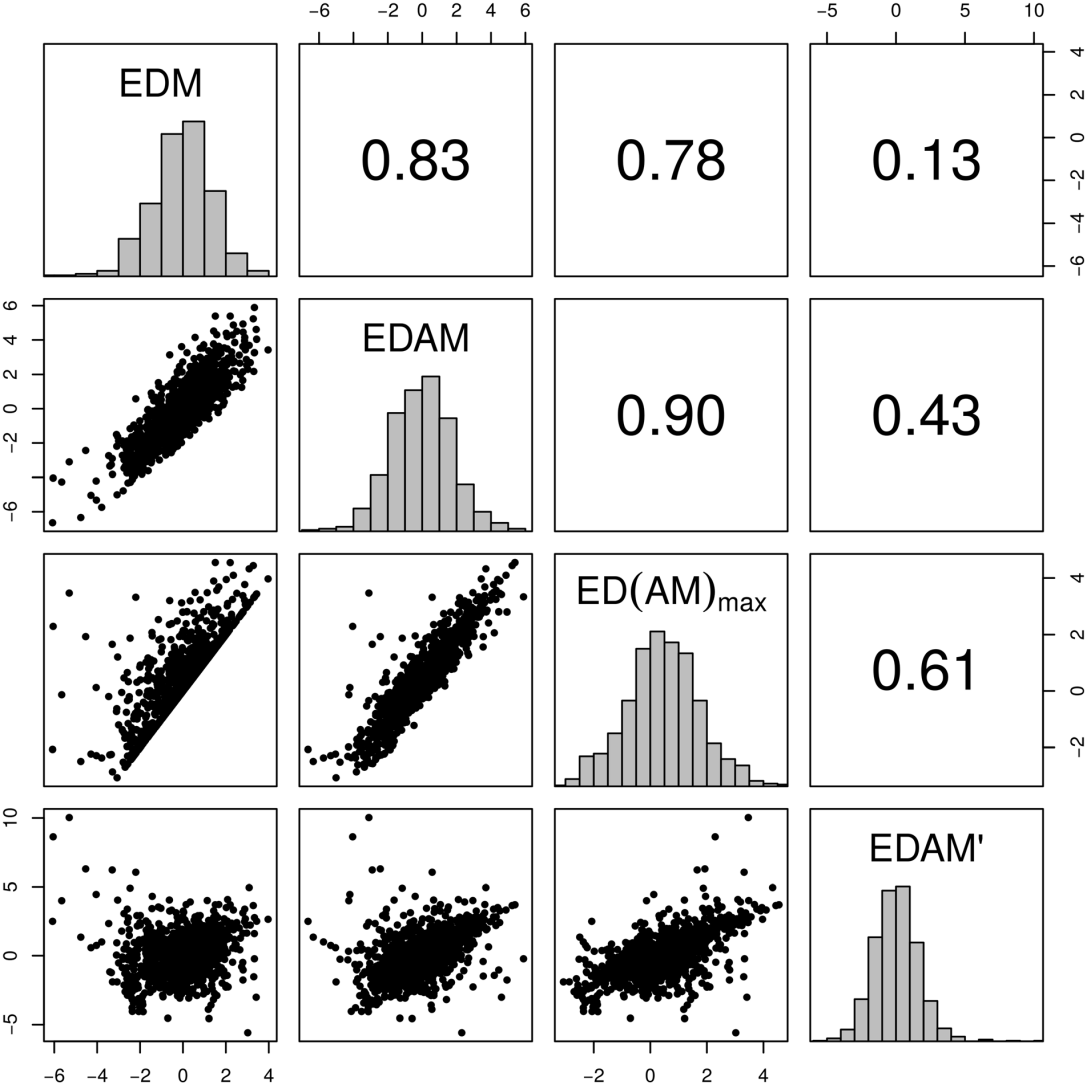
Scatter plots of the *EDAM* measures against each another (lower panels), histograms of their distributions (along the diagonal), and correlation coefficients of the measures (upper panels). Note the poor correlation between *EDAM*′ and *EDM*.

The highest ranking twenty species for each measure are listed in Tables 2 and 3; Fig 4 plots the values of each species under each metric against one-another for comparison. *EDGE* and *EDAM*-family values for all 1060 species in the study are available in S1 File. Although *EDM*, *EDAM*, and *ED*(*AM*)_*max*_ species scores are correlated with one another, *EDAM*′, with its focus on range expansion, highlights a different set of species (Fig 4).

**Table 2:** Species rankings according to *EDGE* and *EDM*.

***EDM***	*EDGE*
*Selaginella selaginoides*	*Lycopodiella inundata*
*Pteridium aquilinum*	*Pilularia globulifera*
*Galeopsis angustifolia*	*Osmunda regalis*
*Pilularia globulifera*	*Selaginella selaginoides*
*Himantoglossum hircinum*	*Hymenophyllum wilsonii*
*Hymenophyllum wilsonii*	*Daphne mezereum*
*Sinapis arvensis*	*Wolffia arrhiza*
*Lycopodiella inundata*	*Isoetes echinospora*
*Botrychium lunaria*	*Isoetes lacustris*
*Osmunda regalis*	*Zostera noltei*
*Ranunculus arvensis*	*Adonis annua*
*Cryptogramma crispa*	*Spartina maritima*
*Huperzia selago*	*Astragalus danicus*
*Dioscorea communis*	*Cuscuta epithymum*
*Hymenophyllum tunbrigense*	*Botrychium lunaria*
*Oxalis acetosella*	*Frankenia laevis*
*Scleranthus annuus*	*Myriophyllum verticillatum*
*Mentha pulegium*	*Colchicum autumnale*
*Narthecium ossifragum*	*Ruppia cirrhosa*
*Tofieldia pusilla*	*Pteridium aquilinum*

Species rankings according to *EDGE* and *EDM*. The highest ranking species is listed first.

**Table 3:** Species rankings according to *EDAM*, *EDAM*′, and *ED*(*AM*)_*max*_.

*EDAM*	*EDAM*′	*ED*(*AM*)_*max*_
*Himantoglossum hircinum*	*Polypodium vulgare*	*Cicendia filiformis*
*Cicendia filiformis*	*Tripleurospermum inodorum*	*Radiola linoides*
*Radiola linoides*	*Polygonum arenastrum*	*Lycopodiella inundata*
*Lycopodiella inundata*	*Polypodium interjectum*	*Osmunda regalis*
*Mentha pulegium*	*Sedum album*	*Zostera marina*
*Zostera marina*	*Osmunda regalis*	*Selaginella selaginoides*
*Galeopsis angustifolia*	*Dryopteris expansa*	*Drosera intermedia*
*Spartina maritima*	*Tripleurospermum maritimum*	*Spartina maritima*
*Cuscuta epithymum*	*Tilia platyphyllos*	*Hymenophyllum tunbridgense*
*Scleranthus annuus*	*Isoetes echinospora*	*Adoxa moschatellina*
*Drosera intermedia*	*Polystichum setiferum*	*Pinguicula lusitanica*
*Pinguicula lusitanica*	*Papaver somniferum*	*Viola lactea*
*Jasione montana*	*Equisetum telmateia*	*Equisetum telmateia*
*Silene gallica*	*Agrostis stolonifera*	*Polypodium vulgare*
*Viola lactea*	*Polystichum aculeatum*	*Cuscuta epithymum*
*Hymenophyllum tunbrigense*	*Adoxa moschatellina*	*Pteridium aquilinum*
*Pteridium aquilinum*	*Vulpia ciliata*	*Galeopsis angustifolia*
*Oxalis acetosella*	*Cicendia filiformis*	*Narthecium ossifragum*
*Adonis annua*	*Radiola linoides*	*Pilularia globulifera*
*Erica tetralix*	*Lycopodiella inundata*	*Himantoglossum hircinum*

Species rankings according to *EDAM*, *EDAM*′, and *ED*(*AM*)_*max*_. The highest ranking species is listed first.

**Fig 5:**
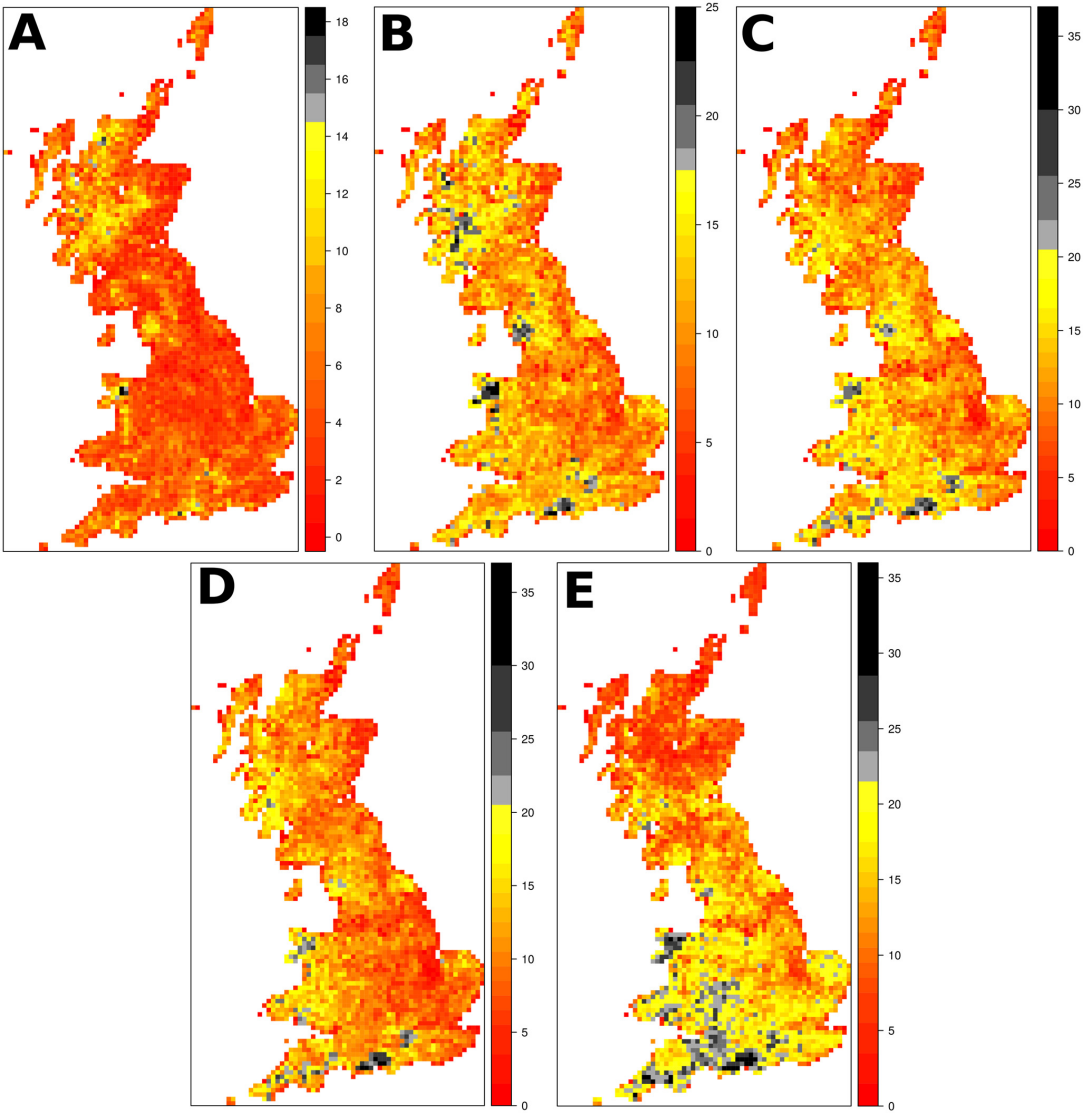
Distribution of top-fifty ranked species across Britain. The sub-panels show *EDGE* (A), *EDM* (B), *ED*(*AM*)_*max*_ (C), *EDAM* (D), and *EDAM*′ (E). Each map has a legend to the right hand side, splitting the counts of species into twenty ‘Jenks’ quantiles (*classIntervals* function in the R package *classInt* [58]). The greatest four quantiles have been coloured differently, to emphasise the high-priority sites.

Maps of the distribution of the top-fifty species for each measure are shown in Fig 5. *EDGE* and *EDM* are correlated (*r* = 0.81, *t*_2811_ = 72.12, *p* < 0.0001); both prioritise the Scottish Highlands, North of Wales, and patches of Northern and Southern England. In general, *EDGE* prioritises more diffuse areas of Britain, while more intense clusters of species are detected with *EDM*. *EDAM* and *ED*(*AM*)_*max*_ highlight similar grid cells to *EDM* (correlations of grid cells’ values; all *r* > = 0.85, *t*_2811_ > = 84.10, *p* < 0.0001). *EDAM*′ places a greater emphasis on England and Wales, prioritising large parts of the South-West and Wales. Maps of the mean scores of the species in each grid cell are shown in the S2 Fig.

## Discussion

### Performance of *EDAM*

*EDM* was more discriminating than *EDGE*, which we suggest means *EDM* provides a more useful prioritisation of British plant species. If species are to be compared and prioritised, it is helpful if the scheme according to which they are compared is unambiguous as to the ranking of those species (there are no tied rankings). We also argue that, since *EDM* gives explicit (and, in our case, equal) weight to both evolutionary distinctiveness and decline, *EDM* gives a clearer prioritisation scheme. It is important to note that rankings mask the magnitude of differences among species; that one species out-ranks another does not mean it does so by much. We would advise against binning *EDGE* and *EDAM* lists (*e.g.*, into ‘highly ranked’ and ‘low-ranked’ species, etc.) where possible since this would remove information from the data. It is better to rank species with more information and simply to be more cautious about closely-ranked species, and if necessary collect more information such as the likelihood of successful conservation intervention (see below).

The data required for the *EDAM* approach means it is unlikely to supplant *EDGE* as a global prioritisation scheme. Predictive accuracy is difficult to quantify, and species distribution models, although tractable and relatively quick to produce, typically ignore biotic interactions [41] and assume species’ distributions are at equilibrium with their environment [31]. However, other methods of assessing predictive accuracy, such as literature reviews and community-based models, can be prohibitively time-consuming and difficult to directly compare among taxa.

*EDM* ranks of some species vary greatly among the bootstrap phylogenies, which is concerning in species where there is no obvious relationship between *EDM* rank and the likelihood of the phylogeny across which it was calculated (Fig 3). In particular, the three distinct groupings in Fig 3b suggest that three islands in treespace were sampled (discussed in [42]); since each island seems equally likely, there may be no correct score for such species. Accurately prioritising species that are difficult to place in a phylogeny (*rogue taxa*; see [43]) without more sequence data may not be possible, potentially affecting the potential value of phylogenetic diversity (see [44]). However, rogue taxa are the exception, not the rule, and on the whole the bootstrap replicates were strongly correlated with the rankings of the best phylogeny. Rogue taxa pose no problem for a prioritisation system as long as they are identified, but testing prioritisation lists by recalculating across candidate trees (as in [5, 45]) will not detect them. Our phylogeny was built using a single DNA locus; future phylogenetic work with more data will likely revise our listing in ways we cannot readily predict or account for with randomisations.

### Prioritisation

At first glance, the number of common species in the lists of prioritised species (e.g., *Pteridium aquilinum*, bracken; table 2) might be surprising. Such surprises reflect how evolutionarily distinct British non-angiosperm plants are relative to each other (*i.e.*, ignoring close relatives outside the UK), but the measures also prioritise several severely declining species (e.g., *Galeopsis angustifolia*—red hemp-nettle). Limiting the lists to angiosperms or down-weighting evolutionary distinctiveness would alter the rankings if desired, and we consider it a strength of the *EDAM* (and *EDGE*) approach that we can make our decisions explicit in this way. More importantly, the purpose of a quantitative prioritisation exercise is not necessarily to produce a single, definitive list for conservation, but to help us consider how we prioritise nature. For example, *Selaginella selaginoides* is fairly uncharismatic (even for a clubmoss), yet it has the highest *EDM* score. This species is declining in Britain, and gives its name to an entire (declining) species group in one text [46], yet is not a UK Biodiversity Action Plan species [47] (and has not been added to the subsequent Section 41 list published under the Natural Environment and Rural Communities Act). *S. selaginoides* is widespread throughout mainland Europe but declining with Britain; these *EDM* rankings re-open the question of whether distinct, declining components of our flora should be conserved regardless of their status elsewhere.

Ours is not the first study to examine the phylogenetic pattern of threat in the UK flora (*e.g.*, [48]), however, we are the first to prioritise species (not clades) and parts of the country. *EDGE* and *EDM* prioritise broadly similar parts of the Britain, but the intensity and resolution of prioritised areas is much greater for *EDM*. This greater intensity likely directly stems from the dependence of *EDM* on range change data and as such is unsurprising, but it shows the *EDAM* approach can give a more precise, and thus feasible, set of geographic priorities. The precautionary *ED*(*AM*)_*max*_ measure seems intermediate between the *EDM* and *EDAM*, which is perhaps to be expected since it is essentially a compound measure, but *EDAM*′ is different in highlighting large parts of southern England. There is potentially a causal link between the high concentration of species with high *EDM* values in the Scottish Highlands (known to species with contracting southern ranges [27]) and the emphasis *EDAM*′ places on Southern England. As with the highlighting of *S. selaginoides*, we suggest this is another example of how systematic prioritisation can (re-)draw attention to potentially important conservation issues.

Given our *EDAM* approach allows for any arbitrary (explicit) relative weighting of its three components, it is reasonable to ask what weighting should be used in practice. We suggest that, while rankings will be sensitive to the weighting used, there is little utility in deciding empirically what the “correct” weighting is. Instead, weighting should be chosen *a priori* based on beliefs and wishes about the importance of species, or chosen as part of an ongoing discussion with stakeholders and the general public. Such an iterative, reflective process should reveal more about what we value about nature, and as such be of greater use than a static list generated by a distant stranger. Conservation prioritisation (triage), and the making of prioritisation lists, is controversial [49, 50]; we argue that the *process* of making lists forces us to confront our prior beliefs with hard data, and under an *EDAM* approach quantify and weight exactly what we value about biodiversity. We feel that weighting everything equally reflects more readily the naïve expectations of someone viewing a ranking, and that is why we have used it. There is no objective criterion by which a person’s subjective beliefs and values can be measured, and open dialogue is the best way to move through disagreement over beliefs. While we have made no attempt to measure the relative costs or likelihood of success of saving these species (*c.f.* [51]), we emphasise that such modifications to the *EDAM* approach are possible. An additional precautionary measure such as *EDAM*(*CS*)_*min*_, where species are additionally ranked according to the *worst* of their cost (C; which should be reversed as we reversed range change) and likelihood of success (S), could make a good continuation of the *EDAM* approach. *EDAM* (and *EDGE*; [52]) exhibit spatial pattern; spatial clumping of threatened species may hinder or help conservation, and this too could be incorporated in future prioritisation schemes.

### Wider conservation issues

There is some evidence that species distantly related to an assemblage are more likely to invade [53], and more damaging when they do [54], although there are counter-examples [55]. More work is needed, but it is reasonable to highlight evolutionarily distinct species with expanding ranges as potential future problems, particularly given that distantly related species tend to be ecologically dissimilar (reviewed in [56]). Although precise ecological data on invasive species are often missing, invasives can usually be placed (perhaps coarsely) within a phylogenetic tree without much difficulty. Thus an *EDAM* approach could help identify potential problems, particularly in concert with information on the phylogenetic structure of protected areas. The UK has excellent data on the species composition of most protected areas, and our phylogeny based upon *rbcL* could be used for such analyses.

Prioritising species on the basis that their declines are poorly-understood might seem an unusual strategy for two reasons. Firstly, such species might be more difficult to save, although this could make them of greater academic interest and investigating them may uncover new conservation techniques. Secondly, it might seem better to weight the decline of a species according to our confidence in that decline, rather than treat magnitude and accuracy as distinct. However, to do so would not necessarily be precautionary (see [57]), and could lead us to prioritise species on the basis of sampling effort. Uncertainty is already enshrined in the Red List criteria [10]; for example, a species qualifies as endangered under criterion A at a lower level of decline if that decline is not understood (and continuing). Accuracy of prediction (ideally) relates to our understanding of the drivers of range change, and, if desired, we could choose to prioritise species with declines we understand well enough to reverse. The *EDAM* framework could be extended to explicitly trade-off the cost of saving species with their distinctiveness or magnitude of threat, along with any other species-specific data a conservation biologist can quantify.

Conservationists can rarely achieve all their goals simultaneously, and instead several components (*e.g.*, evolutionary distinctiveness, species diversity, financial cost, likelihood of success, and ecosystem services) must be traded off against one another. By scaling the components of *EDAM* so that each is on the same scale, we have a starting point from which we can explore the implications of prioritising different aspects of our biota, and make our subjective decisions more explicit. The *EDAM* lists presented here are designed for different purposes, and it is unlikely that a single priority list will ever suffice for British plants, let alone other threatened taxa. However, we feel that the success of the *EDGE* program demonstrates that incorporating evolutionary distinctiveness into conservation strategies strikes a chord with the majority of biologists; the measures we propose here allow uncertainty to be explicitly incorporated as well.

## Supporting Information

S1 Fig Evolutionary distinctiveness is relatively unaffected by the maximum age of genera. Genera were cut into the best-scoring phylogeny as described in the text, but at each integer quantile of genus age, and the evolutionary distinctiveness scores of all species correlated with the scores when the genera were cut at the 1^*st*^ quantile. In the figure, the correlation coefficients are plotted against quantile at which the cuts were made.
(PDF)

S2 Fig Mean of prioritisation measures for species in grid cells across Britain. The sub-panels show *EDGE* (A), *EDM* (B), *ED*(*AM*)_*max*_ (C), *EDAM* (D), and *EDAM*′ (E). Each map has a legend to the right hand side, splitting the values into twenty ‘Jenks’ quantiles (*classIntervals* function in the R package *classInt* [58]). The greatest four quantiles have been coloured differently, to emphasise the high-priority sites. Note that the distributions of all five measures have extremely long tails (as shown by the size of the quantiles in the legends).
(JPG)

S1 File Supporting information for the manuscript, including:

*analysed_accession_numbers.doc*—Accession numbers of sequences used to build phylogeny
*bootstrap_trees.zip*—All bootstrap replicates of phylogeny
*metrics.csv*—CSV file with species scores under all metrics
*novel_sequence_accession_numbers.doc*—Accession numbers of sequences uploaded to GenBank
*OneZoom.html*—Best phylogeny, viewable in OneZoom, embedded in a web page (HTML file)
*phylogeny.tre*—Best dated phylogeny in Newick format
*uncorrected_sequences.fasta*—Uncorrected sequences used in analysis
*uncorrect_vs_corrected_sequence_check.doc*—Demonstration that corrections made to sequences do not affect the results of the manuscript
(ZIP)
