# Supplementary figures and images for: Beyond the EDGE with EDAM: Prioritising British Plant Species According to Evolutionary Distinctiveness, and Accuracy and Magnitude of Decline
Source: PLoS One. 2015 May 27;10(5):e0126524. doi: 10.1371/journal.pone.0126524 (PMC4446313; doi:10.1371/journal.pone.0126524)

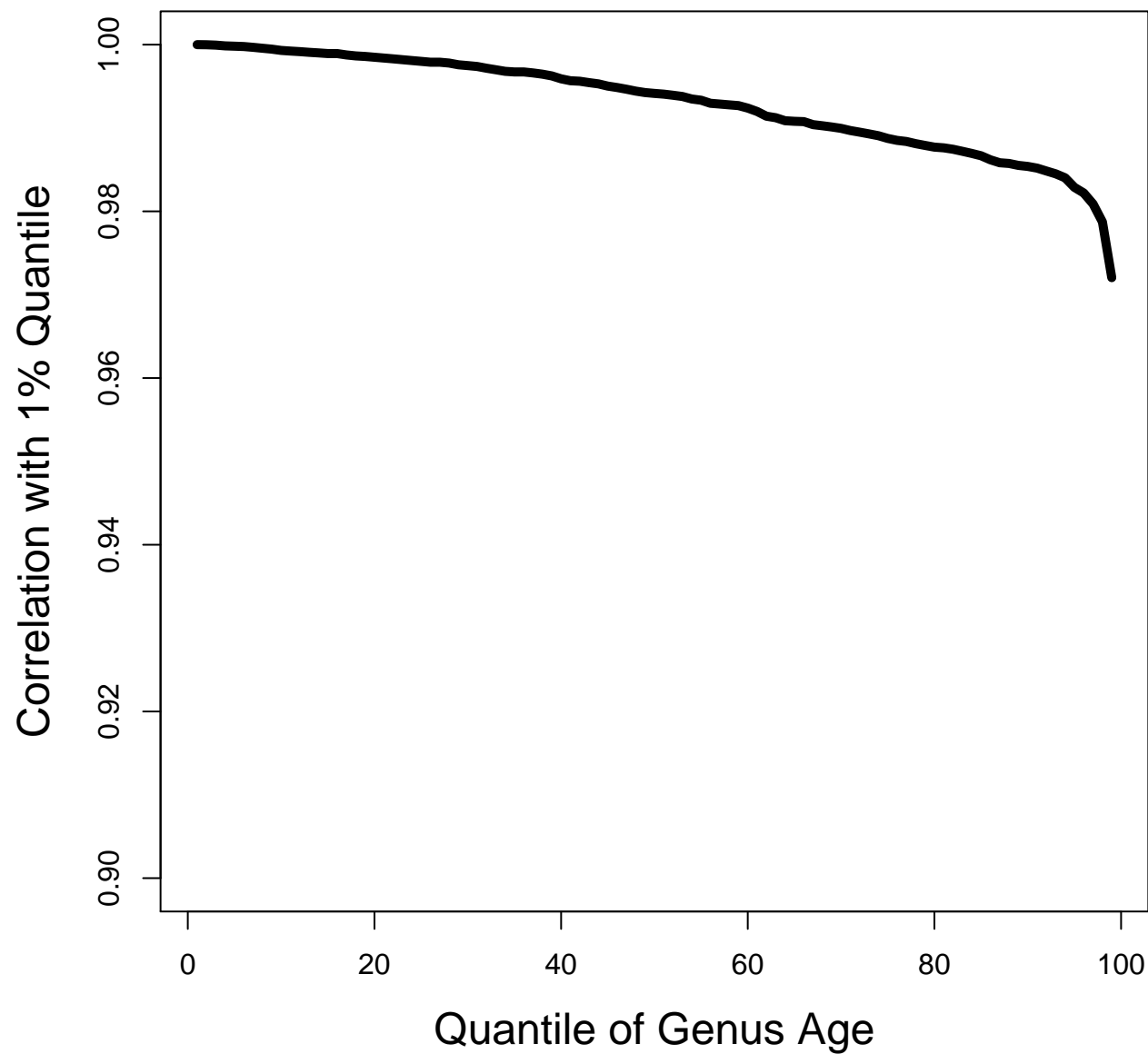

Supplement: S1 Fig — Genera were cut into the best-scoring phylogeny as described in the text, but at each integer quantile of genus age, and the evolutionary distinctiveness scores of all species correlated with the scores when the genera were cut at the 1st quantile. In the figure, the correlation coefficients are plotted against quantile at which the cuts were made. (PDF) [file pone.0126524.s001.pdf]

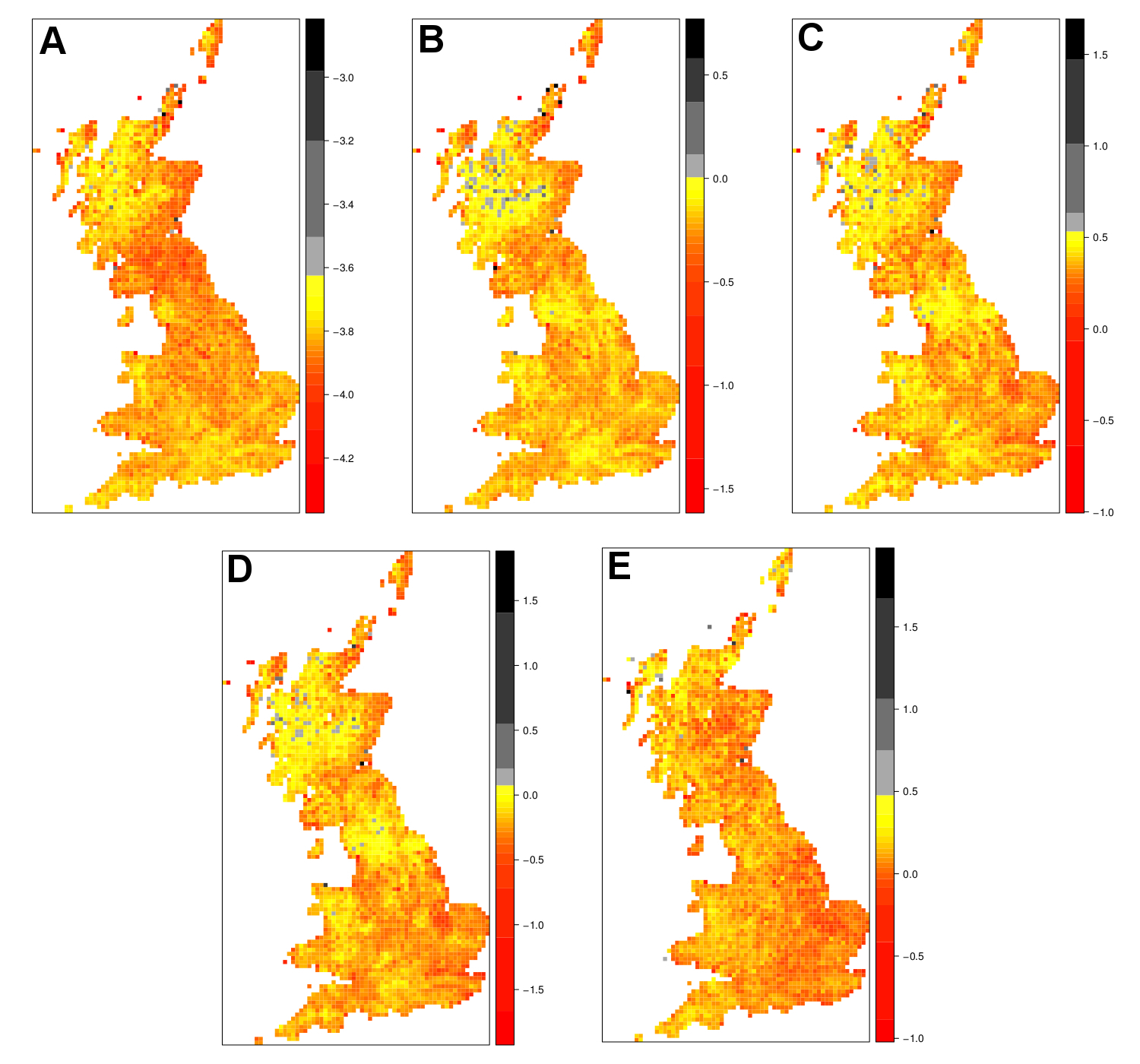

Supplement: S2 Fig — The sub-panels show EDGE (A), EDM (B), ED(AM)max (C), EDAM (D), and EDAM′ (E). Each map has a legend to the right hand side, splitting the values into twenty ‘Jenks’ quantiles (classIntervals function in the R package classInt [58]). The greatest four quantiles have been coloured differently, to emphasise the high-priority sites. Note that the distributions of all five measures have extremely long tails (as shown by the size of the quantiles in the legends). (JPG) [file pone.0126524.s002.jpg]
