# Supplementary material for: Beyond the EDGE with EDAM: Prioritising British Plant Species According to Evolutionary Distinctiveness, and Accuracy and Magnitude of Decline
Source: PLoS One. 2015 May 27;10(5):e0126524. doi: 10.1371/journal.pone.0126524 (PMC4446313; doi:10.1371/journal.pone.0126524)
Supplement: S1 File — Supporting information for the manuscript, including: analysed_accession_numbers.doc—Accession numbers of sequences used to build phylogeny bootstrap_trees.zip—All bootstrap replicates of phylogeny metrics.csv—CSV file with species scores under all metrics novel_sequence_accession_numbers.doc—Accession numbers of sequences uploaded to GenBank OneZoom.html—Best phylogeny, viewable in OneZoom, embedded in a web page (HTML file) phylogeny.tre—Best dated phylogeny in Newick format uncorrected_sequences.fasta—Uncorrected sequences used in analysis uncorrect_vs_corrected_sequence_check.doc—Demonstration that corrections made to sequences do not affect the results of the manuscript (ZIP) [file pone.0126524.s003.zip › Supplementary_Materials/OneZoom.htm]

 OneZoom - Everything on one page, all you have to do is zoom


OneZoom - Tree of Life  
Search
Grow
Options
Data
Reset
Tutorial
More
About
License

Look and Feel 
Detail -
Detail +
Zoom Level
View Type
Colour Scheme
Show Polytomies
 
Beginning
Reverse
Pause
Play
End
Faster
Slower
 
 Search 


 Latin 

 Common 

 Conservation 
Mark Results
Unmark Results
Show Results
Fly to Results
 Data in newick format, ultrametric, polytomies as branch length zero - 

Load data
