# Supplementary material for: Beyond the EDGE with EDAM: Prioritising British Plant Species According to Evolutionary Distinctiveness, and Accuracy and Magnitude of Decline
Source: PLoS One. 2015 May 27;10(5):e0126524. doi: 10.1371/journal.pone.0126524 (PMC4446313; doi:10.1371/journal.pone.0126524)
Supplement: S1 File — Supporting information for the manuscript, including: analysed_accession_numbers.doc—Accession numbers of sequences used to build phylogeny bootstrap_trees.zip—All bootstrap replicates of phylogeny metrics.csv—CSV file with species scores under all metrics novel_sequence_accession_numbers.doc—Accession numbers of sequences uploaded to GenBank OneZoom.html—Best phylogeny, viewable in OneZoom, embedded in a web page (HTML file) phylogeny.tre—Best dated phylogeny in Newick format uncorrected_sequences.fasta—Uncorrected sequences used in analysis uncorrect_vs_corrected_sequence_check.doc—Demonstration that corrections made to sequences do not affect the results of the manuscript (ZIP) [file pone.0126524.s003.zip › Supplementary_Materials/uncorrect_vs_corrected_sequence_check.doc]

**Novel sequences uploaded to GenBank for this project**

As described in the text, minor (single base-pair) mistakes were found at the 3' region in 14 of the 548 sequences produced during this project. Despite not all of those sequences being used in the final analysis, we wanted to ensure that these errors, which have been corrected in the versions uploaded to GenBank, could not have caused any problems with the analyses.

We performed 1000 independent RAxML searches (citation and DNA model options as in the manuscript) on MAFFT (citations and options as in the manuscript) alignments of the corrected and uncorrected sequences. Taking the best (greatest likelihood) tree of each, we calculated the Robinson-Foulds distance between them: it was 0.055684. This compares with a mean distance of 0.099299 and 0.099437 within the uncorrected and corrected tree searches, respectively. Thus the difference *between* the optimal trees found with and without the corrections is lesser than the distances *within* the two datasets, and so these corrections can have had little-to-no effect on the final results.
