# Supplementary material for: Beyond the EDGE with EDAM: Prioritising British Plant Species According to Evolutionary Distinctiveness, and Accuracy and Magnitude of Decline
Source: PLoS One. 2015 May 27;10(5):e0126524. doi: 10.1371/journal.pone.0126524 (PMC4446313; doi:10.1371/journal.pone.0126524)
Supplement: S1 File — Supporting information for the manuscript, including: analysed_accession_numbers.doc—Accession numbers of sequences used to build phylogeny bootstrap_trees.zip—All bootstrap replicates of phylogeny metrics.csv—CSV file with species scores under all metrics novel_sequence_accession_numbers.doc—Accession numbers of sequences uploaded to GenBank OneZoom.html—Best phylogeny, viewable in OneZoom, embedded in a web page (HTML file) phylogeny.tre—Best dated phylogeny in Newick format uncorrected_sequences.fasta—Uncorrected sequences used in analysis uncorrect_vs_corrected_sequence_check.doc—Demonstration that corrections made to sequences do not affect the results of the manuscript (ZIP) [file pone.0126524.s003.zip › Supplementary_Materials/novel_sequence_accession_numbers.doc]

**Novel sequences for this project**

| **Species Name** | **Genbank Accession No.** |
| --- | --- |
| Acaena novae-zelandiae | KM360608 |
| Acer campestre | KM360609 |
| Achillea millefolium | KM360610 |
| Aconitum temulum | KM360611 |
| Acorus calamus | KM360612 |
| Adoxa moscatellina | KM360613 |
| Aegopodium podagraria | KM360614 |
| Aeonium cuneatum | KM360615 |
| Aesculus hippocastanum | KM360616 |
| Aethusa cynapium ssp cynapium | KM360617 |
| Agrostemma githago | KM360618 |
| Ailanthus altissima | KM360619 |
| Aira praecox | KM360620 |
| Alcea rosea | KM360621 |
| Alchemilla filicaulis | KM360622 |
| Alliaria petiolata | KM360623 |
| Allium ursinum | KM360624 |
| Alnus glutinosa | KM360625 |
| Alstroemeria aurea | KM360626 |
| Althaea officinalis | KM360627 |
| Alyssum saxatile | KM360628 |
| Amaranthus retroflexus | KM360629 |
| Amelanchier lamarkii | KM360630 |
| Anchusa arvensis | KM360631 |
| Anemone nemorosa | KM360632 |
| Angelica sylvestris | KM360633 |
| Anisantha sterilis | KM360634 |
| Anthoxanthum odoratum | KM360635 |
| Anthriscus sylvestris | KM360636 |
| Anthyllis vulneraria | KM360637 |
| Aphanes arvensis | KM360638 |
| Apium nodiflorum | KM360639 |
| Aponogeton distachys | KM360640 |
| Aptenia cordifolia | KM360641 |
| Aquilegia vulgaris | KM360642 |
| Arabidopsis thaliana | KM360643 |
| Arabis caucasica | KM360644 |
| Arctium lappa | KM360645 |
| Arctostaphylos alpinus | KM360646 |
| Aremonia agrimonioides | KM360647 |
| Arenaria serpyllifolia | KM360648 |
| Arisarum proboscideum | KM360649 |
| Aristolochia clematitis | KM360650 |
| Armoracia rustica | KM360651 |
| Arrhenatherum elatius | KM360652 |
| Artemisia vulgaris | KM360653 |
| Arum maculatum | KM360654 |
| Asparagus officinalis long | KM360655 |
| Asperula cynanchica | KM360656 |
| Astilbe japonica | KM360657 |
| Astragalus alpinus | KM360658 |
| Atriplex portulacoides | KM360659 |
| Atropa belladonna | KM360660 |
| Aubrieta deltoidea | KM360661 |
| Azolla filiculoides | KM360662 |
| Ballota nigra | KM360663 |
| Barbarea verna | KM360664 |
| Bellis perennis | KM360665 |
| Bergenia crassifolia | KM360666 |
| Berteroa incana | KM360667 |
| Berula erecta | KM360668 |
| Beta vulgaris | KM360669 |
| Betula pendula | KM360670 |
| Bidens tripartita | KM360671 |
| Blackstonia perfoliata | KM360672 |
| Borago officinalis | KM360673 |
| Brachyglottis sunshine | KM360674 |
| Brachypodium sylvaticum | KM360675 |
| Brassica rapa | KM360676 |
| Briza media | KM360677 |
| Bromus erectus | KM360678 |
| Bromus hordeaceus | KM360679 |
| Bryonia dioica | KM360680 |
| Buddleja davidii | KM360681 |
| Bunias orientalis | KM360682 |
| Butomus umbellatus | KM360683 |
| Buxus sempervirens | KM360684 |
| Cakile maritima | KM360685 |
| Calamagrostis x acutifolia | KM360686 |
| Callitriche stagnalis | KM360687 |
| Calluna vulgaris | KM360688 |
| Caltha palustris | KM360689 |
| Campanula glomerata | KM360690 |
| Capsella bursapastoris | KM360691 |
| Cardamine pratensis | KM360692 |
| Carduus nutans | KM360693 |
| Carex panicea | KM360694 |
| Carlina vulgaris | KM360695 |
| Carpinus betula | KM360696 |
| Carprobrotus edulis | KM360697 |
| Carthamus tinctorius | KM360698 |
| Castanea sativa | KM360699 |
| Catabrosa aquatica | KM360700 |
| Cedrus deodara | KM360701 |
| Centaurea nigra | KM360702 |
| Centranthus ruber | KM360703 |
| Cephalaria gigantea | KM360704 |
| Cerastium fontanum | KM360705 |
| Ceratocapnos claviculata | KM360706 |
| Ceratochloa carinata | KM360707 |
| Chaenomeles speciosa | KM360708 |
| Chaenorhinum minor | KM360709 |
| Chaerophyllum temulum | KM360710 |
| Chamaecyparis lawsoni | KM360711 |
| Chamaemelum nobile | KM360712 |
| Chelidonium majus | KM360713 |
| Chenopodium ficifolium | KM360714 |
| Chionodoxa forbesii | KM360715 |
| Choisya ternata | KM360716 |
| Chrysanthemum segetum | KM360717 |
| Chrysosplenium oppositifolium | KM360718 |
| Cicerbita macrophylla | KM360719 |
| Cichorium intybus | KM360720 |
| Circaea lutetiana | KM360721 |
| Cirsium palustre | KM360722 |
| Claytonia sibirica | KM360723 |
| Clematis vitalba | KM360724 |
| Clinopodium vulgare | KM360725 |
| Colutea arborescens | KM360726 |
| Conium maculatum | KM360727 |
| Convallaria majalis | KM360728 |
| Convolvulus arvensis | KM360729 |
| Conyza canadensis | KM360730 |
| Cornus sanguinea | KM360731 |
| Coronopus squamatus | KM360732 |
| Corydalis solida | KM360733 |
| Cotoneaster cambricus | KM360734 |
| Cotula coronopifolia | KM360735 |
| Crasula helmsii | KM360736 |
| Crataegus monogynus | KM360737 |
| Crepis capillaris | KM360738 |
| Crithmum maritimum | KM360739 |
| Crocus vernus | KM360740 |
| Cucubalus baccifer | KM360741 |
| Cyclamen coum | KM360742 |
| Cydonia oblonga | KM360743 |
| Cymbalaria muralis | KM360744 |
| Cynoglossum officinale | KM360745 |
| Cytisus scoparius ssp scoparius | KM360746 |
| Dactylis glomerata | KM360747 |
| Dactylorhiza incarnata ssp cruenta | KM360748 |
| Danthonia decumbens | KM360749 |
| Daphne mezereum | KM360750 |
| Daucus carota | KM360751 |
| Digitalis purpurea | KM360752 |
| Diplotaxis tenuifolia | KM360753 |
| Dipsacus fullonum | KM360754 |
| Doronicum x excelsum | KM360755 |
| Draba incana | KM360756 |
| Dracunculus vulgaris | KM360757 |
| Drosera rotundifolia | KM360758 |
| Duchesnea indica | KM360759 |
| Echinops bannaticus | KM360760 |
| Elaeagnus x ebbingei | KM360761 |
| Eleocharis palustris | KM360762 |
| Elodeae nutallii | KM360763 |
| Elymus caninus | KM360764 |
| Epilobium angustifolium | KM360765 |
| Epimedium pinnatum | KM360766 |
| Eranthis hyemalis | KM360767 |
| Erica cinerea | KM360768 |
| Erigeron glaucus | KM360769 |
| Eriophorum vaginatum | KM360770 |
| Erophila verna | KM360771 |
| Eryngium maritimum | KM360772 |
| Erysimum cheiranthoides | KM360773 |
| Escallonia macrantha | KM360774 |
| Eschscholzia californica | KM360775 |
| Eucalyptus gunnii | KM360776 |
| Euonymus europaeus | KM360777 |
| Euphorbia peplus | KM360778 |
| Euphrasia officinalis | KM360779 |
| Exochorda macrantha | KM360780 |
| Falcaria vulgaris | KM360781 |
| Fallopia convolvulus | KM360782 |
| Fascicularia bicolor | KM360783 |
| Ficus carica | KM360784 |
| Filago vulgaris | KM360785 |
| Filipendula ulmaria | KM360786 |
| Foeniculum vulgare | KM360787 |
| Forsythia X intermedia | KM360788 |
| Fragaria vesca | KM360789 |
| Frangula alnus | KM360790 |
| Fritillaria meleagris | KM360791 |
| Fumaria officinalis | KM360792 |
| Gagea bohemica | KM360793 |
| Galanthus nivalis | KM360794 |
| Galega officinalis | KM360795 |
| Galeopsis bifida | KM360796 |
| Galinsoga parviflora | KM360797 |
| Galium mollugo | KM360798 |
| Gaultheria shallon | KM360799 |
| Genista monspessulana | KM360800 |
| Gentiana pneumonantha | KM360801 |
| Gentianella ciliata | KM360802 |
| Geranium molle | KM360803 |
| Geum rivale | KM360804 |
| Glaucium flavum | KM360805 |
| Glaux maritima | KM360806 |
| Glyceria fluitans | KM360807 |
| Gnaphalium uliginosum | KM360808 |
| Guizotia abyssinnica | KM360809 |
| Hebe Var.‘midsummer beauty’ | KM360810 |
| Helianthemum nummularium | KM360811 |
| Helianthus annuus | KM360812 |
| Hemerocallis fulva | KM360813 |
| Heracleum sphondylium | KM360814 |
| Hesperis matronalis | KM360815 |
| Hieracium chinense name | KM360816 |
| Hippophae rhamnoides | KM360817 |
| Hippuris vulgaris | KM360818 |
| Hirschfeldia incana | KM360819 |
| Holodiscus discolor | KM360820 |
| Homogyne alpina | KM360821 |
| Honckenya peploides | KM360822 |
| Hordeum secalinum | KM360823 |
| Hottonia palustris | KM360824 |
| Humulus lupulus | KM360825 |
| Hyacinthoides nonscripta | KM360826 |
| Hyacinthus orientalis | KM360827 |
| Hypericum humifusum | KM360828 |
| Hypochaeris radicata | KM360829 |
| Iberis sempervirens | KM360830 |
| Ilex aquifolium | KM360831 |
| Illecebrum verticillatum | KM360832 |
| Impatiens parviflora | KM360833 |
| Juncus articulatus | KM360834 |
| Kerria japonica | KM360835 |
| Kniphofia X praecox | KM360836 |
| Laburnum anagyroides | KM360837 |
| Lactuca serriola | KM360838 |
| Lamiastrum galeobdolon | KM360839 |
| Lamium album | KM360840 |
| Lampranthus roseus | KM360841 |
| Lapsana communis | KM360842 |
| Lathyrus pratensis | KM360843 |
| Laurus nobilis | KM360844 |
| Lavandula X intermedia | KM360845 |
| Lavatera arborea | KM360846 |
| Leontodon hispidus | KM360847 |
| Leonurus cardiaca | KM360848 |
| Lepidium heterophyllum | KM360849 |
| Leucanthemum vulgare | KM360850 |
| Leucojum aestivum | KM360851 |
| Ligusticum scoticum | KM360852 |
| Linaria vulgaris | KM360853 |
| Linnaea borealis | KM360854 |
| Linum bienne | KM360855 |
| Liparis loeselii | KM360856 |
| Liriodendron tulipifera | KM360857 |
| Listera ovata | KM360858 |
| Lithodora diffusa cult. heavenlyblue | KM360859 |
| Lloydia serotina | KM360860 |
| Lobularia maritima | KM360861 |
| Loiseleuria procumbens | KM360862 |
| Lolium perenne perenne | KM360863 |
| Lotus corniculatus | KM360864 |
| Luzula campestris | KM360865 |
| Lychnis flos cuculi | KM360866 |
| Lycium chinense | KM360867 |
| Lysichiton americanus | KM360868 |
| Lysimachia nummularia | KM360869 |
| Lythrum salicaria | KM360870 |
| Mahonia aquifolium | KM360871 |
| Malus domestica | KM360872 |
| Malva sylvestris | KM360873 |
| Meconopsis cambrica | KM360874 |
| Medicago lupulina | KM360875 |
| Melampyrum pratens | KM360876 |
| Melica uniflora | KM360877 |
| Melilotus officinalis | KM360878 |
| Melissa officinalis | KM360879 |
| Mercurialis perennis | KM360880 |
| Metasequoia glyptostroboides | KM360881 |
| Meum athamanticum | KM360882 |
| Mimulus moschatus | KM360883 |
| Moehringia trinervia | KM360884 |
| Molinia caerulea | KM360885 |
| Montia fontana chondrosperma | KM360886 |
| Muscari sp. | KM360887 |
| Mycelis muralis | KM360888 |
| Myosotis secunda | KM360889 |
| Myosoton aquaticum | KM360890 |
| Myrica gale | KM360891 |
| Myrrhis odorata | KM360892 |
| Nardus stricta | KM360893 |
| Nepeta X facusesenii | KM360894 |
| Nigella arvensis | KM360895 |
| Nonea lutea | KM360896 |
| Nothofagus antarctica | KM360897 |
| Nymphaea alba | KM360898 |
| Odontites verna | KM360899 |
| Oemleria cerasiformis | KM360900 |
| Oenothera glazioviana | KM360901 |
| Onobrychis sativa | KM360902 |
| Ononis repens | KM360903 |
| Onopordum acanthium | KM360904 |
| Orchis mascula | KM360905 |
| Ornithopus perpusillus | KM360906 |
| Osmanthus minor | KM360907 |
| Osteospermum jucundum | KM360908 |
| Ostrya carpinifolia | KM360909 |
| Oxyria digyna | KM360910 |
| Oxytropis campestris | KM360911 |
| Pachysandra terminalis | KM360912 |
| Paeonia officinalis | KM360913 |
| Panicum miliaceum | KM360914 |
| Parentucellia viscosa | KM360915 |
| Parietaria judaica | KM360916 |
| Paris quadrifolia | KM360917 |
| Parthenocissus inserta | KM360918 |
| Pastinaca sativa | KM360919 |
| Pedicularis sylvatica | KM360920 |
| Pentaglottis sempervirens | KM360921 |
| Persicaria amphibia | KM360922 |
| Petasites hybridus | KM360923 |
| Petrorhagia sp | KM360924 |
| Peucedanum palustre | KM360925 |
| Phalaris arundinacea | KM360926 |
| Phleum pratense ssp | KM360927 |
| Phlomis fruticosa | KM360928 |
| Photinia x fraseri | KM360929 |
| Phragmites australis | KM360930 |
| Phygelius capensis | KM360931 |
| Physocarpus opulifolius | KM360932 |
| Picris echioides | KM360933 |
| Pimpinella saxifraga | KM360934 |
| Pinguicula vulgaris | KM360935 |
| Poa pratensis | KM360936 |
| Polemonium caerulium | KM360937 |
| Polygonatum multiflorum | KM360938 |
| Polypogon monspeliensis | KM360939 |
| Populus nigra | KM360940 |
| Prunella vulgaris | KM360941 |
| Prunus spinosa | KM360942 |
| Pseudofumaria lutea | KM360943 |
| Pseudotsuga menziesii | KM360944 |
| Pterocarya fraxinifolia | KM360945 |
| Pulicaria dysenteria | KM360946 |
| Pulmonaria officinalis | KM360947 |
| Pulsatilla vulgaris | KM360948 |
| Pyracantha coccinea | KM360949 |
| Pyrola minor | KM360950 |
| Pyrus pyraster | KM360951 |
| Radiola linoides | KM360952 |
| Ranunculus acris | KM360953 |
| Reseda lutea | KM360954 |
| Rhamnus cathartica | KM360955 |
| Rhinanthus minor | KM360956 |
| Rhododendron ponticum | KM360957 |
| Ribes rubrum | KM360958 |
| Robinia pseudoacacia | KM360959 |
| Rosmarinus officinalis | KM360960 |
| Rubus idaeus | KM360961 |
| Rumex acetosa | KM360962 |
| Salicornia europaea | KM360963 |
| Salvia pratensis | KM360964 |
| Samolus valerandi | KM360965 |
| Sanguisorba officinalis | KM360966 |
| Santolina chamaecyparsissas | KM360967 |
| Saponaria officinalis | KM360968 |
| Sarcococca confusa | KM360969 |
| Saussurea alpina | KM360970 |
| Saxifraga aizoides | KM360971 |
| Scabiosa columbaria | KM360972 |
| Scandix pecten veneris | KM360973 |
| Schoenus nigricans | KM360974 |
| Scilla verna | KM360975 |
| Scirpus sylvatica | KM360976 |
| Scutellaria galericulata | KM360977 |
| Securigera varia | KM360978 |
| Sedum rosea | KM360979 |
| Senecio aquaticus | KM360980 |
| Silene maritima | KM360981 |
| Sison amomum | KM360982 |
| Sisymbrium officinale | KM360983 |
| Sium latifolium | KM360984 |
| Smyrnium perfoliatum | KM360985 |
| Solanum dulcamara | KM360986 |
| Soleirolia soleirolii | KM360987 |
| Solidago canadensis | KM360988 |
| Sonchus oleraceus | KM360989 |
| Sorbus domestica | KM360990 |
| Sorghum halepense | KM360991 |
| Sparganium erectum | KM360992 |
| Spartium junceum | KM360993 |
| Spergula arvensis | KM360994 |
| Spiraea x arguta | KM360995 |
| Spirodela polyrhiza | KM360996 |
| Stachys sylvatica | KM360997 |
| Stellaria graminea | KM360998 |
| Stratiotes aloides | KM360999 |
| Subularia aquatica | KM361000 |
| Symphoricarpos X cheanaultii | KM361001 |
| Syringa vulgaris | KM361002 |
| Tamarix gallica | KM361003 |
| Tanacetum vulgare | KM361004 |
| Taraxacum officinale agg | KM361005 |
| Taxodium distichum | KM361006 |
| Tellima grandiflora | KM361007 |
| Tetragonolobus maritimus | KM361008 |
| Teucrium scorodonia | KM361009 |
| Thalictrum aquilegiifolium | KM361010 |
| Thesium humifusum | KM361011 |
| Thlaspi arvense | KM361012 |
| Thymus polytrichus | KM361013 |
| Tilia X europaea | KM361014 |
| Tofieldia pusilla | KM361015 |
| Torilis japonica | KM361016 |
| Trachystemon orientalis | KM361017 |
| Tragopogon pratensis | KM361018 |
| Trientalis europaea | KM361019 |
| Trifolium repens | KM361020 |
| Triglochin maritimum | KM361021 |
| Tripleurospermum inodorum | KM361022 |
| Trisetum flavescens | KM361023 |
| Tussilago farfara | KM361024 |
| Ulex europaeus | KM361025 |
| Ulmus glabra | KM361026 |
| Urtica urens | KM361027 |
| Vaccinium myrtillus | KM361028 |
| Valerianella locusta | KM361029 |
| Verbascum virgatum | KM361030 |
| Verbena officinalis | KM361031 |
| Vicia cracca | KM361032 |
| Vinca minor | KM361033 |
| Viola arvensis | KM361034 |
| Vulpia bromoides | KM361035 |
| Waldsteinia ternata | KM361036 |
| Weigela florida | KM361037 |
| Yucca recurvifolia | KM361038 |
| Zannichellia palustris | KM361039 |
| Zizania latifolia | KM361040 |
| Zostera angustifolia | KM361041 |
