# Supplementary material for: Beyond the EDGE with EDAM: Prioritising British Plant Species According to Evolutionary Distinctiveness, and Accuracy and Magnitude of Decline
Source: PLoS One. 2015 May 27;10(5):e0126524. doi: 10.1371/journal.pone.0126524 (PMC4446313; doi:10.1371/journal.pone.0126524)
Supplement: S1 File — Supporting information for the manuscript, including: analysed_accession_numbers.doc—Accession numbers of sequences used to build phylogeny bootstrap_trees.zip—All bootstrap replicates of phylogeny metrics.csv—CSV file with species scores under all metrics novel_sequence_accession_numbers.doc—Accession numbers of sequences uploaded to GenBank OneZoom.html—Best phylogeny, viewable in OneZoom, embedded in a web page (HTML file) phylogeny.tre—Best dated phylogeny in Newick format uncorrected_sequences.fasta—Uncorrected sequences used in analysis uncorrect_vs_corrected_sequence_check.doc—Demonstration that corrections made to sequences do not affect the results of the manuscript (ZIP) [file pone.0126524.s003.zip › Supplementary_Materials/analysed_accession_numbers.doc]

**Sequences used duing phylogenetic analysis**

As described in the text, several specimens were collected and sequences but not used in the phylogeny of this manuscript. Their sequences, listed in 'novel_sequence_accession_numbers.doc', have been released onto GenBank as a courtesy to the field. Below are the sequences, and their accession numbers, used for all the analyses in this project.

| **Genus (tip label in phylogeny)** | **Genbank Accession No.** |
| --- | --- |
| abies | AF456380 |
| abutilon | HM849734 |
| acaena | KM360608 |
| acanthus | L12592 |
| acer | KM360609 |
| aceras | AF074203 |
| achillea | KM360610 |
| aconitum | KM360611 |
| acorus | KM360612 |
| actaea | FJ626588 |
| adiantum | D14880 |
| adonis | AY954487 |
| adoxa | KM360613 |
| aegopodium | KM360614 |
| aesculus | KM360616 |
| aethusa | KM360617 |
| agrimonia | U06791 |
| agrostemma | KM360618 |
| agrostis | EF115543 |
| aira | KM360620 |
| ajuga | Z37385 |
| alchemilla | KM360622 |
| alisma | L08759 |
| alliaria | KM360623 |
| allium | KM360624 |
| alnus | KM360625 |
| alopecurus | AJ746281 |
| alstroemeria | KM360626 |
| althaea | KM360627 |
| alyssum | KM360628 |
| amaranthus | KM360629 |
| amelanchier | KM360630 |
| ammophila | AJ784835 |
| anagallis | M88343 |
| anchusa | KM360631 |
| andromeda | AF124572 |
| anemanthele | EF125158 |
| anemone | KM360632 |
| angelica | KM360633 |
| anogramma | AF336102 |
| antennaria | HE574602 |
| anthemis | HM849779 |
| anthoxanthum | KM360635 |
| anthriscus | KM360636 |
| anthyllis | KM360637 |
| antirrhinum | L11688 |
| apera | EF125147 |
| aphanes | KM360638 |
| apium | KM360639 |
| aquilegia | KM360642 |
| arabidopsis | U91966 |
| arabis | KM360643 |
| araucaria | U96467 |
| arbutus | L12597.2 |
| arctium | KM360645 |
| arctostaphylos | KM360646 |
| arenaria | KM360648 |
| armeria | Z97640 |
| armoracia | KM360651 |
| arrhenatherum | KM360652 |
| artemisia | KM360653 |
| arum | KM360654 |
| asparagus | KM360655 |
| asperula | KM360656 |
| asplenium | AB574874 |
| aster | GQ436492 |
| astragalus | KM360658 |
| athyrium | U05908 |
| atriplex | KM360659 |
| atropa | KM360660 |
| aubrieta | KM360661 |
| avena | L15300 |
| avenula | EF125144 |
| azolla | KM360662 |
| baldellia | U80677 |
| ballota | KM360663 |
| barbarea | KM360664 |
| bartsia | AF190903 |
| beckmannia | EF125142 |
| bellis | KM360665 |
| berberis | AF139878 |
| bergenia | KM360666 |
| berula | KM360668 |
| beta | KM360669 |
| betula | KM360670 |
| bidens | KM360671 |
| blackstonia | KM360672 |
| blechnum | U62037 |
| blysmus | AJ404700 |
| bolboschoenus | Y12996 |
| borago | KM360673 |
| botrychium | L13474 |
| brachypodium | KM360675 |
| brassica | KM360676 |
| briza | KM360677 |
| bromus | KM360679 |
| bryonia | KM360680 |
| buddleja | KM360681 |
| bupleurum | U50224 |
| butomus | KM360683 |
| buxus | KM360684 |
| cakile | KM360685 |
| calamagrostis | KM360686 |
| calendula | AB530953 |
| callistephus | AB530954 |
| callitriche | KM360687 |
| calluna | KM360688 |
| caltha | KM360689 |
| calystegia | AY100992 |
| campanula | KM360690 |
| capsella | KM360691 |
| cardamine | KM360692 |
| carduus | KM360693 |
| carex | KM360694 |
| carlina | KM360695 |
| carpinus | KM360696 |
| carpobrotus | KM360697 |
| castanea | KM360699 |
| catabrosa | KM360700 |
| catapodium | EF125150 |
| cedrus | KM360701 |
| celastrus | AJ235775 |
| centaurea | KM360702 |
| centaurium | HM849878 |
| centranthus | KM360703 |
| cephalanthera | AF074123 |
| cerastium | KM360705 |
| ceratocapnos | KM360706 |
| ceratophyllum | M77030 |
| cercis | Z70163 |
| chaenorhinum | KM360709 |
| chaerophyllum | KM360710 |
| chamaecyparis | KM360711 |
| chamaemelum | KM360712 |
| chamerion | L10217 |
| chelidonium | KM360713 |
| chenopodium | KM360714 |
| chimonobambusa | JN247205 |
| chrysanthemum | KM360717 |
| chrysocoma | AM234859 |
| chrysosplenium | KM360718 |
| cicendia | HM849894 |
| cicer | AF308707 |
| cicerbita | KM360719 |
| cichorium | KM360720 |
| cicuta | D44561 |
| circaea | KM360721 |
| cirsium | KM360722 |
| cladium | Y12988 |
| claytonia | KM360723 |
| clematis | KM360724 |
| clinopodium | KM360725 |
| cochlearia | FN594827 |
| colchicum | L12673.2 |
| colutea | KM360726 |
| conium | KM360727 |
| consolida | FJ626582 |
| convallaria | KM360728 |
| convolvulus | KM360729 |
| conyza | HM849911 |
| corallorhiza | JF319717 |
| coriandrum | L11676 |
| cornus | KM360731 |
| coronopus | KM360732 |
| corrigiola | FN868311 |
| cortaderia | AJ746260 |
| corylus | X56619 |
| cosmos | AB530960 |
| cotinus | AY510148 |
| cotoneaster | KM360734 |
| crassula | KM360736 |
| crataegus | KM360737 |
| crepis | KM360738 |
| crinum | HM640488 |
| crithmum | KM360739 |
| crocosmia | AJ577250 |
| cruciata | X81097 |
| cryptogramma | DQ432660 |
| cupressus | L12571.2 |
| cuscuta | AJ320208 |
| cymbalaria | KM360744 |
| cynodon | AM235055 |
| cynoglossum | KM360745 |
| cynoglottis | EU599890 |
| cynosurus | HM849932 |
| cyperus | AF449509 |
| cypripedium | AF074142 |
| cystopteris | U05916 |
| cytisus | KM360746 |
| daboecia | L12611.2 |
| dactylis | KM360747 |
| dactylorhiza | KM360748 |
| dahlia | AB530962 |
| damasonium | U80678 |
| danthonia | KM360749 |
| daphne | KM360750 |
| datura | U08611 |
| daucus | KM360751 |
| deschampsia | AY691635 |
| descurainia | FN594838 |
| dianthus | M77699 |
| diapensia | L12612.2 |
| digitalis | KM360752 |
| digitaria | AJ746264 |
| diphasiastrum | L11055 |
| diplotaxis | KM360753 |
| dipsacus | KM360754 |
| disphyma | AM234789 |
| dittrichia | HM849957 |
| doronicum | KM360755 |
| draba | KM360756 |
| drosanthemum | HM849959 |
| drosera | KM360758 |
| dryas | U59818 |
| dryopteris | U05923 |
| dysphania | HM587599 |
| ecballium | AF534746 |
| echinochloa | AM887871 |
| echinops | KM360760 |
| echium | EU599874 |
| elaeagnus | KM360761 |
| elatine | AJ402948 |
| eleocharis | KM360762 |
| elodea | KM360763 |
| elymus | KM360764 |
| elytrigia | Z49846 |
| empetrum | AF421091 |
| epilobium | KM360765 |
| epipactis | Z73707 |
| equisetum | GU191334 |
| eranthis | KM360767 |
| erica | KM360768 |
| erigeron | KM360769 |
| eriocaulon | L10252.2 |
| eriophorum | KM360770 |
| erodium | L14694.2 |
| erophila | KM360771 |
| eryngium | KM360772 |
| erysimum | KM360773 |
| escallonia | KM360774 |
| eschscholzia | KM360775 |
| eucalyptus | KM360776 |
| euonymus | KM360777 |
| eupatorium | L13649 |
| euphorbia | KM360778 |
| euphrasia | KM360779 |
| fagopyrum | AB000320 |
| fagus | L13340.2 |
| fallopia | KM360782 |
| ficaria | EU053919 |
| ficus | KM360784 |
| filago | KM360785 |
| filipendula | KM360786 |
| foeniculum | KM360787 |
| forsythia | KM360788 |
| fragaria | KM360789 |
| frangula | HM850010 |
| frankenia | Z97638 |
| fraxinus | DQ673301 |
| fritillaria | KM360791 |
| fuchsia | L10220 |
| fumaria | KM360792 |
| gagea | KM360793 |
| galanthus | KM360794 |
| galega | KM360795 |
| galeopsis | KM360796 |
| galinsoga | KM360797 |
| galium | KM360798 |
| gaudinia | HM850023 |
| gaultheria | KM360799 |
| gazania | L13638 |
| genista | KM360800 |
| gentiana | KM360801 |
| gentianella | KM360802 |
| geranium | KM360803 |
| geum | KM360804 |
| gladiolus | AF206772 |
| glaucium | KM360805 |
| glaux | KM360806 |
| glechoma | L14292 |
| glyceria | KM360807 |
| gnaphalium | KM360808 |
| goodyera | AF074174 |
| groenlandia | U80720 |
| gymnocarpium | U05925 |
| hedera | L01924.2 |
| helenium | GU817764 |
| helianthemum | KM360811 |
| helianthus | KM360812 |
| helleborus | AY954485 |
| helminthotheca | HM850259 |
| hemerocallis | KM360813 |
| heracleum | KM360814 |
| herniaria | AF132091 |
| hesperis | KM360815 |
| heuchera | L01925.2 |
| hieracium | KM360816 |
| hierochloe | AY691634 |
| himantoglossum | AY368337 |
| hippocrepis | Z70169 |
| hippophae | KM360817 |
| hippuris | KM360818 |
| hirschfeldia | KM360819 |
| holcus | AM235062 |
| homogyne | KM360821 |
| honckenya | KM360822 |
| hordelymus | EU376159 |
| hordeum | KM360823 |
| hornungia | DQ310538 |
| hottonia | KM360824 |
| humulus | KM360825 |
| huperzia | Y07934 |
| hyacinthoides | KM360826 |
| hydrilla | AB004891 |
| hydrocharis | U80701 |
| hydrocotyle | U50236 |
| hymenophyllum | AF275648 |
| hyoscyamus | HQ216126 |
| hypericum | KM360828 |
| hypochaeris | KM360829 |
| iberis | KM360830 |
| ilex | KM360831 |
| illecebrum | KM360832 |
| impatiens | KM360833 |
| inula | AY874438 |
| iris | L05037.2 |
| isatis | FN594830 |
| isoetes | AJ010855 |
| isolepis | Y12962 |
| jasione | EU713387 |
| juglans | KM360945 |
| juncus | KM360834 |
| juniperus | L12573.2 |
| kalmia | KM360862 |
| kerria | KM360835 |
| kickxia | HM850096 |
| knautia | Y10698 |
| kobresia | U49232 |
| koeleria | AJ746262 |
| koenigia | AF297144 |
| laburnum | KM360837 |
| lactuca | KM360838 |
| lagarosiphon | U80703 |
| lamium | KM360840 |
| lapsana | KM360842 |
| larix | X63663 |
| lathraea | AF026833 |
| lathyrus | KM360843 |
| laurus | KM360844 |
| lavandula | KM360845 |
| leersia | U13228 |
| legousia | EU713367 |
| lemna | M91630 |
| leontodon | KM360847 |
| lepidium | KM360849 |
| leucanthemum | KM360850 |
| leucojum | KM360851 |
| leycesteria | AJ420872 |
| leymus | Z49843 |
| ligusticum | KM360852 |
| ligustrum | L11686 |
| lilium | AB034929 |
| limonium | Y16904 |
| linaria | KM360853 |
| linnaea | KM360854 |
| linum | KM360855 |
| liparis | KM360856 |
| lithospermum | AM234928 |
| littorella | HM850128 |
| lloydia | KM360860 |
| lobelia | L13930 |
| lobularia | KM360861 |
| lolium | KM360863 |
| lonicera | AJ420870 |
| lotus | KM360864 |
| ludwigia | L10222 |
| lupinus | Z70054 |
| luronium | U80680 |
| luzula | KM360865 |
| lychnis | KM360866 |
| lycium | KM360867 |
| lycopodiella | AJ133261 |
| lycopodium | Y07936 |
| lycopus | HM850150 |
| lysichiton | KM360868 |
| lysimachia | KM360869 |
| lythrum | KM360870 |
| mahonia | KM360871 |
| maianthemum | D17380 |
| malus | KM360872 |
| malva | KM360873 |
| marrubium | Z37412 |
| matricaria | HE574591 |
| matteuccia | D43917 |
| matthiola | HM850161 |
| mauranthemum | AB530971 |
| meconopsis | KM360874 |
| medicago | KM360875 |
| melampyrum | KM360876 |
| melica | KM360877 |
| melilotus | KM360878 |
| melissa | KM360879 |
| mentha | U28876 |
| menyanthes | L14006.2 |
| mercurialis | KM360880 |
| mertensia | EU599837 |
| meum | KM360882 |
| mimulus | KM360883 |
| misopates | HM850180 |
| moehringia | KM360884 |
| molinia | KM360885 |
| montia | KM360886 |
| muehlenbeckia | HM850184 |
| muscari | KM360887 |
| mycelis | KM360888 |
| myosotis | KM360889 |
| myosoton | KM360890 |
| myosurus | DQ099441 |
| myrica | KM360891 |
| myriophyllum | L11195.2 |
| myrrhis | KM360892 |
| najas | U03731 |
| narcissus | AF116972 |
| nardus | KM360893 |
| narthecium | AJ286560 |
| nassella | EF125159 |
| nasturtium | HM850197 |
| neottia | AY368364 |
| nepeta | KM360894 |
| nigella | KM360895 |
| nuphar | DQ069501 |
| nymphaea | KM360898 |
| nymphoides | X87392 |
| odontites | KM360899 |
| oemleria | KM360900 |
| oenanthe | D44577 |
| oenothera | KM360901 |
| onobrychis | KM360902 |
| onoclea | U05936 |
| ononis | KM360903 |
| onopordum | KM360904 |
| ophioglossum | AF313582 |
| ophrys | AF074202 |
| orchis | KM360905 |
| oreopteris | HQ676506 |
| origanum | Z37427 |
| ornithogalum | HM850214 |
| ornithopus | KM360906 |
| orthilia | AF419838 |
| oryzopsis | HQ590194 |
| osmunda | AB076259 |
| oxalis | L01938.2 |
| oxyria | KM360910 |
| oxytropis | KM360911 |
| paeonia | KM360913 |
| pancratium | HE603957 |
| panicum | KM360914 |
| papaver | L08764 |
| parapholis | EF125154 |
| parentucellia | KM360915 |
| parietaria | KM360916 |
| paris | KM360917 |
| parnassia | L01939.2 |
| parthenocissus | KM360918 |
| paspalum | HM850238 |
| pastinaca | KM360919 |
| pedicularis | KM360920 |
| pentaglottis | KM360921 |
| pericallis | HM850241 |
| persicaria | KM360922 |
| petasites | KM360923 |
| petrorhagia | KM360924 |
| petroselinum | AY188433 |
| peucedanum | KM360925 |
| phalaris | KM360926 |
| phegopteris | AF425111 |
| philadelphus | AF323195 |
| phleum | KM360927 |
| phlox | F206809 |
| phormium | Z69232 |
| phragmites | KM360930 |
| phyllodoce | U49292 |
| phymatosorus | EU482960 |
| physalis | U08617 |
| phyteuma | EU713361 |
| picea | AB045039 |
| picris | KM360933 |
| pilularia | U24263 |
| pimpinella | KM360934 |
| pinguicula | KM360935 |
| pinus | AY115763 |
| pisum | AF308705 |
| plantago | L36454 |
| platanthera | AF074215 |
| plecostachys | AM234902 |
| poa | KM360936 |
| polemonium | KM360937 |
| polycarpon | AF132090 |
| polygala | AM234193 |
| polygonatum | KM360938 |
| polygonum | AF297127 |
| polypodium | AY362604 |
| polypogon | KM360939 |
| polystichum | AF537261 |
| populus | KM360940 |
| potamogeton | U344674 |
| potentilla | HM850287 |
| poterium | AY395560 |
| primula | AF394982 |
| prunella | KM360941 |
| prunus | KM360942 |
| pseudofumaria | KM360943 |
| pseudotsuga | KM360944 |
| pteridium | U05940 |
| pterocarya | U00439 |
| puccinellia | L14621 |
| pulicaria | KM360946 |
| pulmonaria | KM360947 |
| pulsatilla | KM360948 |
| pyracantha | KM360949 |
| pyrola | KM360950 |
| pyrus | KM360951 |
| quercus | M58391 |
| radiola | KM360952 |
| ranunculus | KM360953 |
| raphanus | HM849821 |
| reseda | KM360954 |
| rhamnus | KM360955 |
| rhinanthus | KM360956 |
| rhododendron | KM360957 |
| rhus | U00440 |
| rhynchospora | Y12977 |
| ribes | KM360958 |
| robinia | KM360959 |
| romulea | AJ309659 |
| rorippa | AF020328 |
| rosa | U06824 |
| rosmarinus | KM360960 |
| rubia | X83666 |
| rubus | KM360961 |
| rumex | KM360962 |
| ruppia | U03729 |
| ruschia | AM234795 |
| ruscus | Z77274 |
| sagina | HM850329 |
| sagittaria | L08767 |
| salicornia | KM360963 |
| salix | AJ235793 |
| salsola | HM131786 |
| salvia | KM360964 |
| sambucus | AJ420867 |
| samolus | KM360965 |
| sanguisorba | KM360966 |
| sanicula | L11170 |
| santolina | KM360967 |
| saponaria | KM360968 |
| sarcocornia | AY270126 |
| sasa | AJ746278 |
| saussurea | KM360970 |
| saxifraga | KM360971 |
| scabiosa | KM360972 |
| scandix | KM360973 |
| scheuchzeria | U03728 |
| schoenoplectus | Y12943 |
| schoenus | KM360974 |
| scilla | KM360975 |
| scirpoides | Y12994 |
| scirpus | KM360976 |
| scleranthus | AY270145 |
| scorzonera | GQ436482 |
| scrophularia | L36449 |
| scutellaria | KM360977 |
| secale | AY836181 |
| securigera | U74222 |
| sedum | KM360979 |
| selaginella | AF419048 |
| senecio | KM360980 |
| sequoia | L25755.2 |
| sesleria | EF125156 |
| setaria | EF125138 |
| sherardia | X81106 |
| sibthorpia | HM850352 |
| sigesbeckia | GQ436446 |
| silene | KM360981 |
| silybum | HM850356 |
| simethis | Z69231 |
| sinapis | X73284 |
| sison | KM360982 |
| sisymbrium | KM360983 |
| sisyrinchium | Z77290 |
| sium | KM360984 |
| smyrnium | KM360985 |
| solanum lycopersicon | L14403 |
| soleirolia | KM360987 |
| solidago | KM360988 |
| soliva | HM850371 |
| sonchus | KM360989 |
| sorbus | KM360990 |
| sparganium | KM360992 |
| spartina | AM849382 |
| spergula | KM360994 |
| spergularia | HM850381 |
| spiraea | KM360995 |
| spiranthes | AF074229 |
| spirodela | KM360996 |
| stachys | KM360997 |
| staphylea | AJ238406 |
| stellaria | KM360998 |
| stratiotes | KM360999 |
| suaeda | FJ436010 |
| subularia | KM361000 |
| sutera | AM235149 |
| symphoricarpos | KM361001 |
| symphytum | EU599878 |
| syringa | KM361002 |
| tamarix | KM361003 |
| tamus | AF307474 |
| tanacetum | KM361004 |
| taraxacum | KM361005 |
| taxus | AF456388 |
| teesdalia | HE616650 |
| tellima | KM361007 |
| tephroseris | GU817786 |
| teucrium | KM361009 |
| thalictrum | KM361010 |
| thelypteris | U05947 |
| thesium | KM361011 |
| thlaspi | KM361012 |
| thuja | AY237154 |
| thymus | KM361013 |
| tilia | KM361014 |
| tofieldia | KM361015 |
| tolmiea | U06223 |
| torilis | KM361016 |
| tradescantia | L05041.2 |
| tragopogon | KM361018 |
| trichomanes | AF275651 |
| trichophorum | Y12969 |
| trientalis | KM361019 |
| trifolium | KM361020 |
| triglochin | KM361021 |
| tripleurospermum | KM361022 |
| trisetum | KM361023 |
| tristagma | Z69206 |
| triteleia | JQ273934 |
| triticum | EU492898 |
| trollius | AY954486 |
| tropaeolum | L14706.2 |
| tsuga | AY056581 |
| tuberaria | AJ233120 |
| tussilago | KM361024 |
| typha | L05464 |
| ulex | KM361025 |
| ulmus | KM361026 |
| umbilicus | HM850434 |
| urtica | KM361027 |
| utricularia | L13190.2 |
| vaccinium | KM361028 |
| valeriana | L13934 |
| valerianella | KM361029 |
| verbascum | KM361030 |
| verbena | KM361031 |
| veronica | AY034021 |
| viburnum | L13928 |
| vicia | KM361032 |
| vinca | KM361033 |
| viola | KM361034 |
| viscum | L26078 |
| vitis | L01960.2 |
| vulpia | KM361035 |
| wahlenbergia | AM234947 |
| wolffia | AY034254 |
| woodsia | AB021726 |
| zannichellia | KM361039 |
| zea | X86563.2 |
| zostera | KM361041 |
